# Occurrence of Far-Red Light Photoacclimation (FaRLiP) in Diverse Cyanobacteria

**DOI:** 10.3390/life5010004

**Published:** 2014-12-29

**Authors:** Fei Gan, Gaozhong Shen, Donald A. Bryant

**Affiliations:** 1 Department of Biochemistry and Molecular Biology, The Pennsylvania State University, University Park, PA 16802, USA; E-Mails: fxg142@psu.edu (F.G.); gxs22@psu.edu (G.S.); 2 Department of Chemistry and Biochemistry, Montana State University, Bozeman, MT 59717, USA

**Keywords:** photosynthesis, photoacclimation, chlorophyll *d*, chlorophyll *f*, photosystem, phycobilisomes, far-red light, light harvesting

## Abstract

Cyanobacteria have evolved a number of acclimation strategies to sense and respond to changing nutrient and light conditions. *Leptolyngbya* sp. JSC-1 was recently shown to photoacclimate to far-red light by extensively remodeling its photosystem (PS) I, PS II and phycobilisome complexes, thereby gaining the ability to grow in far-red light. A 21-gene photosynthetic gene cluster (*rfpA*/*B*/*C*, *apcA2*/*B2*/*D2*/*E2*/*D3*, *psbA3*/*D3*/*C2*/*B2*/*H2*/*A4*, *psaA2*/*B2*/*L2*/*I2*/*F2*/*J2*) that is specifically expressed in far-red light encodes the core subunits of the three major photosynthetic complexes. The growth responses to far-red light were studied here for five additional cyanobacterial strains, each of which has a gene cluster similar to that in *Leptolyngbya* sp. JSC-1. After acclimation all five strains could grow continuously in far-red light. Under these growth conditions each strain synthesizes chlorophylls *d*, *f* and *a* after photoacclimation, and each strain produces modified forms of PS I, PS II (and phycobiliproteins) that absorb light between 700 and 800 nm. We conclude that these photosynthetic gene clusters are diagnostic of the capacity to photoacclimate to and grow in far-red light. Given the diversity of terrestrial environments from which these cyanobacteria were isolated, it is likely that FaRLiP plays an important role in optimizing photosynthesis in terrestrial environments.

## 1. Introduction

Chlorophototrophic bacteria, *i.e.*, bacteria that use bacteriochlorophylls and/or chlorophylls (Chls) to produce energy for growth, are a diverse group of organisms that currently occur in seven phyla: *Cyanobacteria*, *Proteobacteria*, *Chlorobi*, *Chloroflexi*, *Firmicutes*, *Acidobacteria*, and *Gemmatimonadetes* [1,2,3]. Cyanobacteria are unique among chlorophototrophs by virtue of their ability to photo-oxidize water and evolve oxygen, a reaction that has globally important consequences. Oxygenic photosynthesis was responsible for the oxygenation of Earth’s atmosphere 2.4 Gya [4,5], an event that dramatically altered evolution of life on Earth, and CO_2_ fixation and the accompanying oxygen evolution by cyanobacteria are still essential geochemical processes today. Marine cyanobacteria, mostly *Prochlorococcus*, marine *Synechococcus*, and *Trichodesmium* species, are presently estimated to contribute ≥25% of primary production on Earth [6,7]. Reliable values for cyanobacterial primary production in terrestrial environments are more difficult to estimate, but cyanobacteria are thought to be major contributors to both terrestrial photosynthesis and nitrogen fixation [7,8]. One study suggested that total terrestrial photosynthesis by cyanobacteria is likely to be greater than half, and perhaps equivalent to, their activity in oceans [7]. Thus, cyanobacterial photosynthesis could globally account for as much as half the CO_2_ fixation and oxygen evolution on Earth.

Cyanobacteria principally employ three large, multisubunit complexes to harvest light energy and transduce it into stored chemical energy, principally as ATP and NADPH, for CO_2_ and N_2_ fixation. Photosystem (PS) I acts as a cytochrome *c*_6_ (plastocyanin): ferredoxin oxidoreductase by producing a weak oxidant (P700^+^) and a strong reductant (F_B_^−^) [9]. Cyanobacterial PS I typically occurs as a trimeric complex, in which each monomer contains 11 or 12 polypeptide subunits, 96 Chl *a* molecules, 22 β–carotenes, 2 phylloquinones and 3 [4Fe-4S] centers [10,11]. PS II acts a water:plastoquinone oxidoreductase by producing a powerful oxidant (P680^+^) and a weak reductant (reduced plastoquinone, PQH_2_) [12]. PS II in cyanobacteria mostly occurs in thylakoids as dimers, in which each monomer comprises ~20 polypeptide subunits, 35 Chl *a*, 11 β–carotenes, 2 plastoquinones, 2 hemes, 1 non-heme Fe atom, 4 Mn atoms, and 3–4 Ca atoms [13,14]. The light-harvesting antenna complexes in cyanobacteria are phycobilisomes (PBS), which are very large (~4.5 to 8 MDa) complexes composed of brilliantly colored, water-soluble phycobiliproteins and associated linker polypeptides for their assembly [15]. Allophycocyanin (λ_max_ ~650 nm), phycocyanin (λ_max_ ~620 nm), phycoerythrin ((λ_max_ ~560 nm) and phycoerythrocyanin ((λ_max_ ~575 nm) are the most common phycobiliproteins. Minor allophycocyanin-related proteins (e.g., ApcD, ApcE, and ApcF) play important roles in organization of the core substructure and in the energy transfer and distribution to PS I and PS II [15,16,17].

Because light has both beneficial and deleterious effects on chlorophototrophs, cyanobacteria have evolved mechanisms to cope with excess irradiation and to optimize light harvesting when diffuse, low-light conditions limit growth. Cyanobacteria can alter their total Chl and PBS content; adjust their PS I to PS II ratio; perform non-photochemical quenching using the orange carotenoid protein; and modify their light-harvesting complexes in response to nutrient stresses (S, N, Fe limitation [18,19,20,21,22,23]). One of the best-characterized acclimative responses in cyanobacteria is Complementary Chromatic Acclimation (CCA), a phenomenon discovered more than 100 years ago [24,25,26,27]. In response to green light (GL) or red light (RL), CCA results in remodeling of the peripheral rods of PBS so that they absorb light complementary to the incident radiation. In GL, phycoerythrin is the predominant component of the peripheral rods and cells appear brownish in color. However, phycoerythrin is replaced by phycocyanin in RL to optimize light harvesting, and cells appear blue-green in color [26,27,28]. CCA is principally controlled by a two-component, phosphorelay regulatory system, which is produced from three genes: *rcaC*, *rcaE*, and *rcaF* [26,27]. RcaE is a knotted phytochrome photosensor that is a RL/GL-responsive histidine kinase; RcaF is a small CheY-like signal-receiver/response regulator; and RcaC is the key transcriptional regulator, which has three CheY-like signal receiver domains as well as a winged-helix, DNA-binding domain [26,27].

*Leptolyngbya* sp. JSC-1 (hereafter *Leptolyngbya* JSC-1) is a filamentous, non-heterocystous cyanobacterium that was isolated from a floating mat associated with a thermal feature at LaDuke Hot Springs near Gardiner, MT [29]. *Leptolyngbya* JSC-1 exhibits a number of interesting acclimative responses, both to light wavelength as well as nutrient depletion (e.g., Fe) [28,29,30]. An examination of the *Leptolyngbya* JSC-1 genome suggested that this organism should be capable of type-III CCA, and this property has been confirmed by growing cells in RL and GL (see [28,30]). Remarkably, however, *Leptolyngbya* JSC-1 also exhibits an extensive acclimative response to far-red light (FRL; 700 nm ≤ λ ≤ 800 nm). Under FRL growth conditions, the core subunits of PS I, PS II, and PBS are replaced by the products of a 21-gene cluster, the products of which are specifically expressed in FRL [28]. This acclimation response is apparently controlled by a light-wavelength-dependent, two-component regulatory system: RfpA, RfpB, and RfpC. RfpA is a red/far-red photosensor that belongs to a specific structural group of knotless phytochromes. RfpC is a CheY-like response regulator, and RfpB is a response regulator with two CheY-like signal receiver domains that flank a DNA-binding domain. In addition to Chl *a*, cells grown in FRL synthesize Chls *f* and *d*, and as a result of these and other changes, all three photosynthetic complexes exhibit enhanced absorption between 700 and 800 nm [28]. Moreover, >40% of the genes in the JSC-1 genome show altered transcript abundance 24 h after a shift from white light (WL) to FRL. Relative transcript abundances increase at least 2-fold for ~900 genes and decrease at least 50% for ~2000 genes. Compared to cells grown in RL, *Leptolyngbya* JSC-1 cells grown in FRL exhibit oxygen evolution rates that are ~40% greater than those of cells grown in RL, when FRL is used the actinic light [28]. The combined impact of all of these changes is that *Leptolyngbya* JSC-1 cells can grow continuously and photoautotrophically in FRL—conditions that do not support the growth of most cyanobacteria, including the common model organisms *Synechococcus* sp. PCC 7002 or *Synechocystis* sp. PCC 6803. This acclimative response, denoted Far-Red Light Photoacclimation (FaRLiP), is likely to be important in terrestrial environments in which FRL predominates. These include benthic environments, dense microbial mats, surface-associated cyanobacterial blooms, soil, shade and any other environments where light is strongly filtered by Chl *a* [28].

Among cyanobacteria whose genomes have presently been sequenced, twelve strains have gene clusters similar to the 21-gene cluster expressed specifically in FRL in *Leptolyngbya* JSC-1 (see Figure S3 in [28]). In the present study, we tested whether the presence of this 21-gene cluster, and the characteristics of the genes contained within it, could be considered to be diagnostic of the capacity to grow in FRL, to synthesize Chl *f* and Chl *d*, and to produce PS I, PS II, and PBS complexes with absorption between 700 and 800 nm [28]. We specifically tested five additional strains for these properties and found that all five have the capacity to photoacclimate to and grow in FRL. Given the range of habitats from which the tested strains were isolated, the results suggest that FaRLiP is likely to be widespread among terrestrial cyanobacteria and thus is likely to be an important determinant of terrestrial primary productivity.

## 2. Results

**Figure 1:**
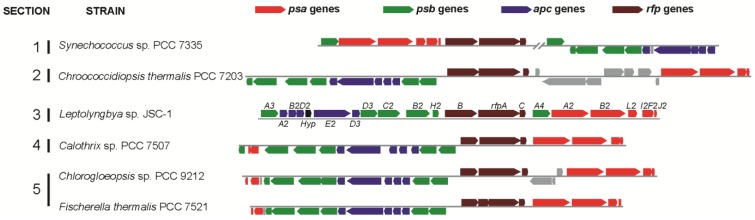
Gene clusters similar to the 21-gene cluster in *Leptolyngbya* JSC-1 occur in five other cyanobacteria that belong to all five sections of the phylum Cyanobacteria. Color-coding for genes: *psa* genes for core subunits of PS I (red); *psb* genes for core subunits of PS II (green); *apc* genes for core subunits of the PBS (blue); *rfp* genes for the knotless phytochrome (RfpA) and response regulators RfpB and RfpC (brown); and conserved hypothetical protein (Hyp, black). All other genes unrelated to photosynthesis are shown in gray. Locus designations are shown for the genes from *Leptolyngbya* JSC-1. The *rfpA* gene in *Fischerella thermalis* is divided into two open reading frames because of sequencing or assembly errors.

Gan *et al.* [28] found that the *Leptolyngbya* JSC-1 genome encodes paralogous genes for the core subunits of PS I, PS II, and PBS that are specifically expressed in FRL. These genes are clustered together with genes encoding a knotless phytochrome (RfpA), a CheY-like signal receiver (RfpC), and a DNA-binding response regulator (RfpB) in a 21-gene cluster (Figure 1). Figure 1 shows that similar gene clusters occur in five additional cyanobacterial strains studied here: *Synechococcus* sp. PCC 7335, *Chroococcidiopsis thermalis* PCC 7203, *Calothrix* sp. PCC 7507, *Fisherella thermalis* PCC 7521, and *Chlorogloeopsis* sp. PCC 9212. Compared to *Leptolyngbya* JSC-1, some of the clusters in these organisms contain additional genes or have undergone rearrangements, but the clusters in all five organisms have orthologs of 17 genes (*psaA2*, *psaB2*, *psaI2*, *psaL2*, *psaF2*, *psaJ2*, *psbA3*, *psbA4*, *psaB2*, *psbC2*, *psbD3*, *psbH2*, *apcA2*, *apcB2*, *apcD3*, *apcD4*, and *apcE2*), which encode core subunits of the three major photosynthetic complexes that are expressed and used during phototrophic growth in FRL [28]. We hypothesized that this gene cluster is diagnostic of cells that can perform FaRLiP, and we further predicted that each of these five strains would undergo similar photoacclimative changes as *Leptolyngbya* JSC-1 in response to exposure to FRL.

**Figure 2:**
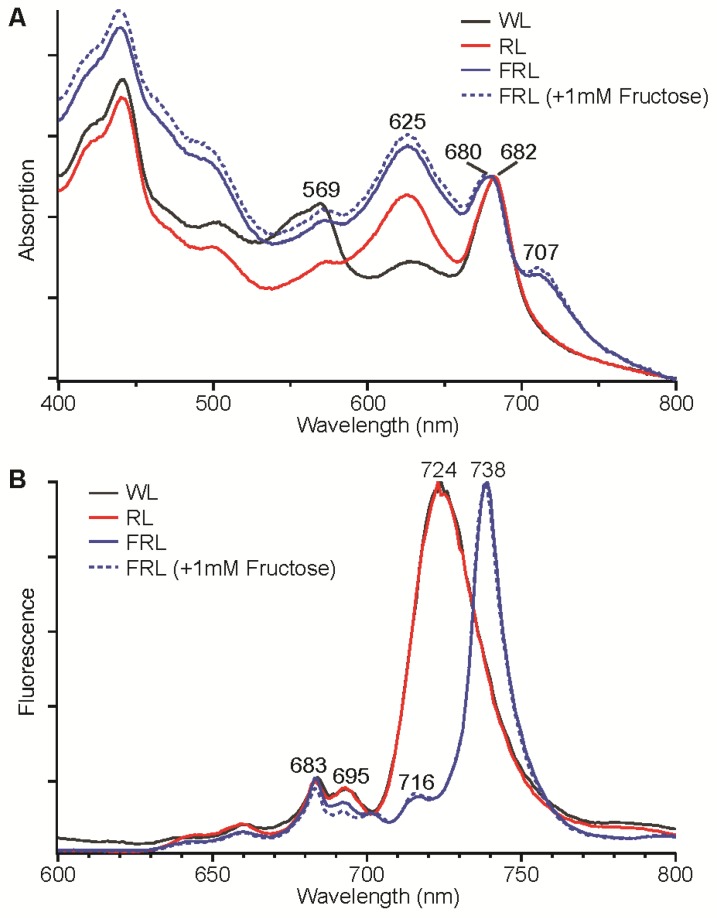
Absorption spectra (**A**) and low-temperature (77 K) fluorescence emission spectra (**B**) for *Synechococcus* sp. PCC 7335 cells grown in white light (WL, solid black line), red light (RL, solid red line), far-red light (FRL, solid blue line), and FRL with 1 mM fructose added to the growth medium (dotted blue line). The excitation wavelength was 440 nm for the fluorescence emission spectra in panel B.

Like *Leptolyngbya* JSC-1, *Synechococcus* sp. PCC 7335 is a phycoerythrin-producing strain that is known to perform type-III CCA [31,32]. To determine whether this marine cyanobacterium could also perform FaRLiP, cells were grown under different light conditions. Figure 2A shows whole-cell absorption spectra for *Synechococcus* sp. PCC 7335 cells that had been grown in WL, 645-nm RL, and 720-nm FRL (±1 mM fructose). The absorption spectrum of whole cells grown in WL had much higher absorption at ~569 nm due to phycoerythrin and much less absorption at 625 nm compared to cells grown in 645-nm RL. These cells were brown and blue-green in color, respectively, which is consistent with previous studies that have shown that *Synechococcus* sp. PCC 7335 is capable of Type-III CCA [31,32]. Cells grown in 720-nm FRL, with or without 1 mM fructose present, had very similar absorption spectra (Figure 2A). The blue-green colored cells exhibited an absorption feature extending from 700 nm to 800 nm with a maximum at ~707 nm that was not observed for cells grown under WL and 645-nm RL conditions. Figure 2B shows the low-temperature fluorescence emission spectra of the same *Synechococcus* sp. PCC 7335 cells. Cells grown in WL and 645-nm RL had nearly identical fluorescence emission spectra, which exhibited maxima at 683 and 695 nm for PS II and 724 nm for PS I. Cells grown in 720-nm FRL (± 1 mM fructose) had a very sharp and intense fluorescence emission maximum at 738 nm with lesser maxima at 716 nm and 683 nm and rather minimal emission at 695 nm. These fluorescence emission properties are similar to those *Leptolyngbya* JSC-1 cells producing Chl *d* and Chl *f*, except that the major Chl emission peak is blue-shifted about 10 nm [28]. These absorption and fluorescence emission results indicate that *Synechococcus* sp. PCC 7335 synthesizes different pigments and/or assembles different photosynthetic complexes with absorption beyond 700 nm when cells are grown in FRL.

**Figure 3:**
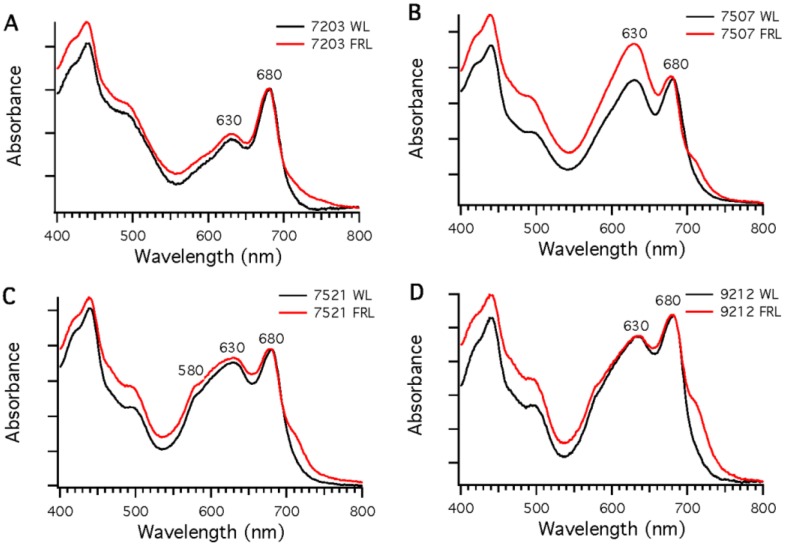
Comparison of whole-cell absorption spectra of cells grown in white light (WL; black lines) or far-red light (FRL; red lines). Each spectrum is the average of three measurements. To facilitate comparisons, the spectra were normalized at 680 nm. (**A**) *Chr. thermalis* PCC 7203; (**B**) *Calothrix* sp. PCC 7507; (**C**) *F. thermalis* PCC 7521; (**D**) *Chlorogloeopsis* sp. PCC 9212.

Four additional strains (*Chr. thermalis* PCC 7203, *Calothrix* sp. PCC 7507, *F. thermalis* PCC 7521, and *Chlorogloeopsis* sp. PCC 9212), all of which have photosynthetic gene clusters similar to that in *Leptolyngbya* JSC-1 (Figure 1), were also grown in FRL to determine if they too could perform FaRLiP. None of these four strains can synthesize phycoerythrin, and thus none of these strains is capable of CCA. However, biochemical studies [32] and genome analyses indicate that all four of these strains can synthesize phycoerythrocyanin. All four strains could be grown photoautotrophically in FRL. Figure 3 shows absorption spectra of whole cells of these strains after growth in WL or 720-nm FRL. In each case, the spectra show enhanced absorption beyond 700 nm in cells that had been grown in FRL, which indicates that new pigments and/or photosynthetic complexes had been synthesized.

**Figure 4:**
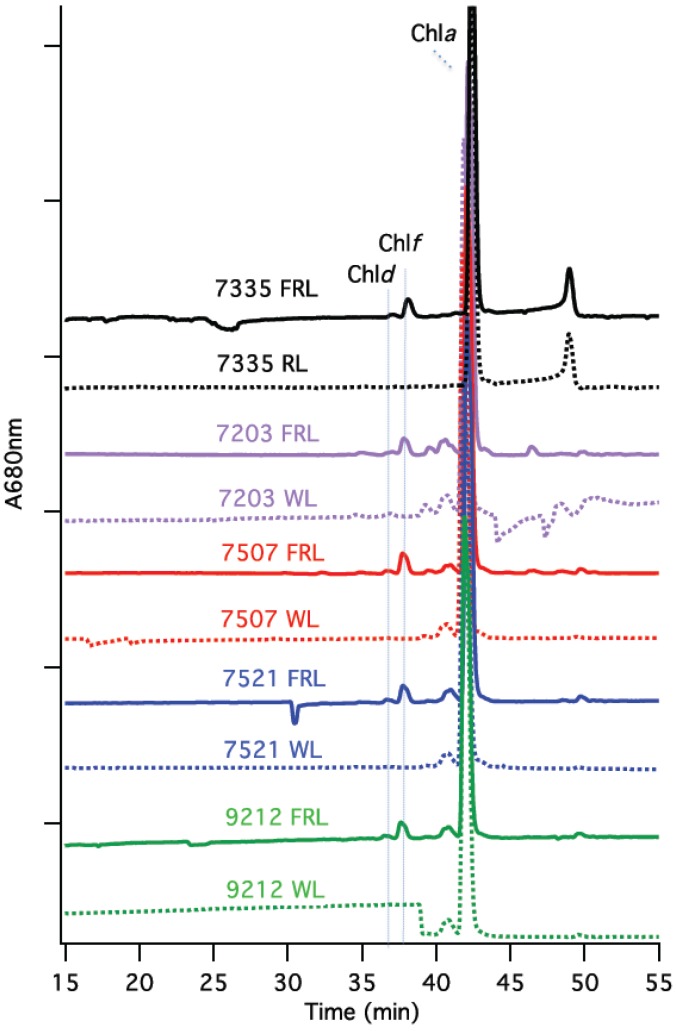
HPLC chromatograms of the Chls extracted from cells grown in white light (WL) and far-red light (FRL). Pigments were extracted and analyzed by reversed-phase HPLC for *Synechococcus* sp. PCC 7335 (7335), *Chr. thermalis* PCC 7203 (7203), *Calothrix* sp. PCC 7507 (7507), *F. thermalis* sp. PCC 7521 (7521), and *Chlorogloeopsis* sp. PCC 9212 (9212). The elution profile at 680 nm is plotted; Chls *a*, *d*, and *f* have similar molar absorptivity values at this wavelength. Chl *d* eluted at about 37 min, Chl *f* at about 38 min, and Chl *a* at about 43 min under the conditions employed.

**Figure 5:**
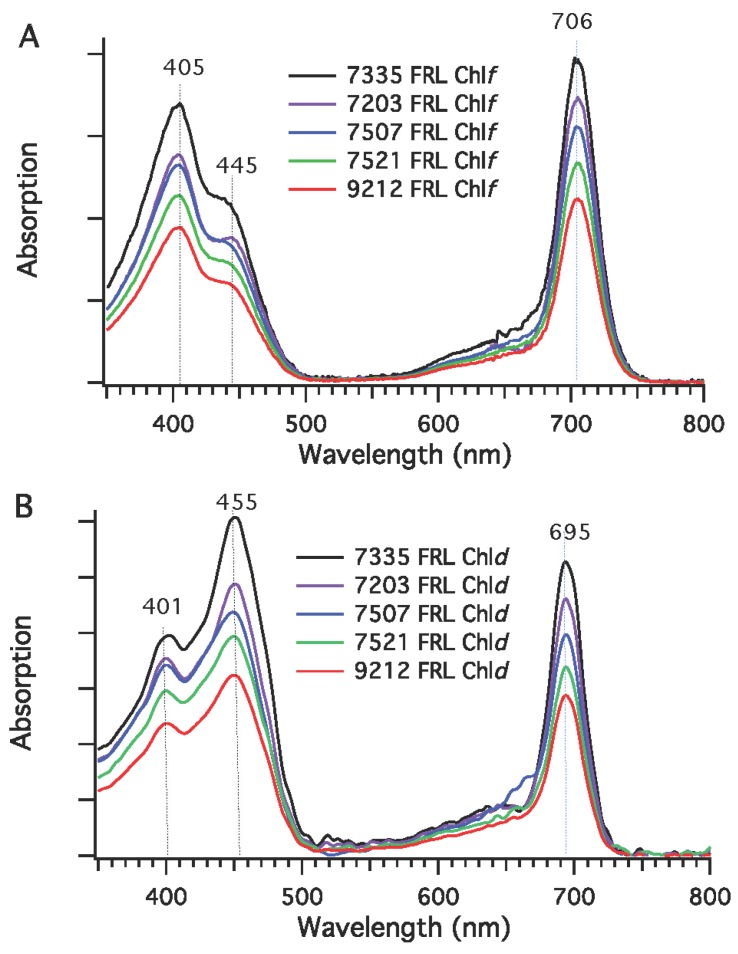
In-line absorption spectra for Chl *f* and Chl *d* from five cyanobacterial strains grown in far-red light (FRL). (**A**) Absorption spectra of Chl *f*; (**B**) Absorption spectra of Chl *d.* Chls were extracted from cells of *Synechococcus* sp. PCC 7335 (7335), *Chr. thermalis* PCC 7203 (7203), *Calothrix* sp. PCC 7507 (7507), *F. thermalis* PCC 7521 (7521), and *Chlorogloeopsis* sp. PCC 9212 (9212), which had been grown in FRL.

Reversed-phase HPLC analyses were conducted to identify pigments extracted from cells of *Synechococcus* sp. PCC 7335, *F. thermalis* PCC 7521, *Calothrix* sp. PCC 7507, *Chlorogloeopsis* sp. PCC 9212, and *C. thermalis* PCC 7203 that had been grown in WL (or 645-nm RL for *Synechococcus* sp. PCC 7335) and FRL. As shown in Figure 4, only Chl *a* was detected in cells grown in WL (or RL). However, two additional peaks that eluted earlier than Chl *a* (at 43 min) were detected in the HPLC profiles of the Chls extracted from cells grown in FRL. The elution times (37 and 38 min) for these minor Chls exactly matched those of Chl *d* and Chl *f*, respectively, which had previously been identified and verified by mass spectrometry for *Leptolyngbya* JSC-1 [28]. As confirmed by the in-line absorption spectra (Figure 5), the Q_y_ absorption maximum of Chl *f* occurred at 706 nm and the Soret maxima were observed at 405 nm and 445 nm (Figure 5A) [28,33]. The in-line absorption spectrum of Chl *d* exhibited a Q_y_ absorption maximum at 695 nm and had Soret absorption maxima at 401 nm and 455 nm (Figure 5B) [28,33]. The amounts of Chl *d* and *f* seemed to vary somewhat under the growth conditions employed. Chl *d* was estimated to be ~1% to 2% of the total Chl, and Chl *f* accounted for about 5% to 10% of the total Chl produced by these strains. These results establish that the biosynthesis of Chl *f* and Chl *d* is probably a property shared by all cyanobacteria that perform FaRLiP.

**Figure 6:**
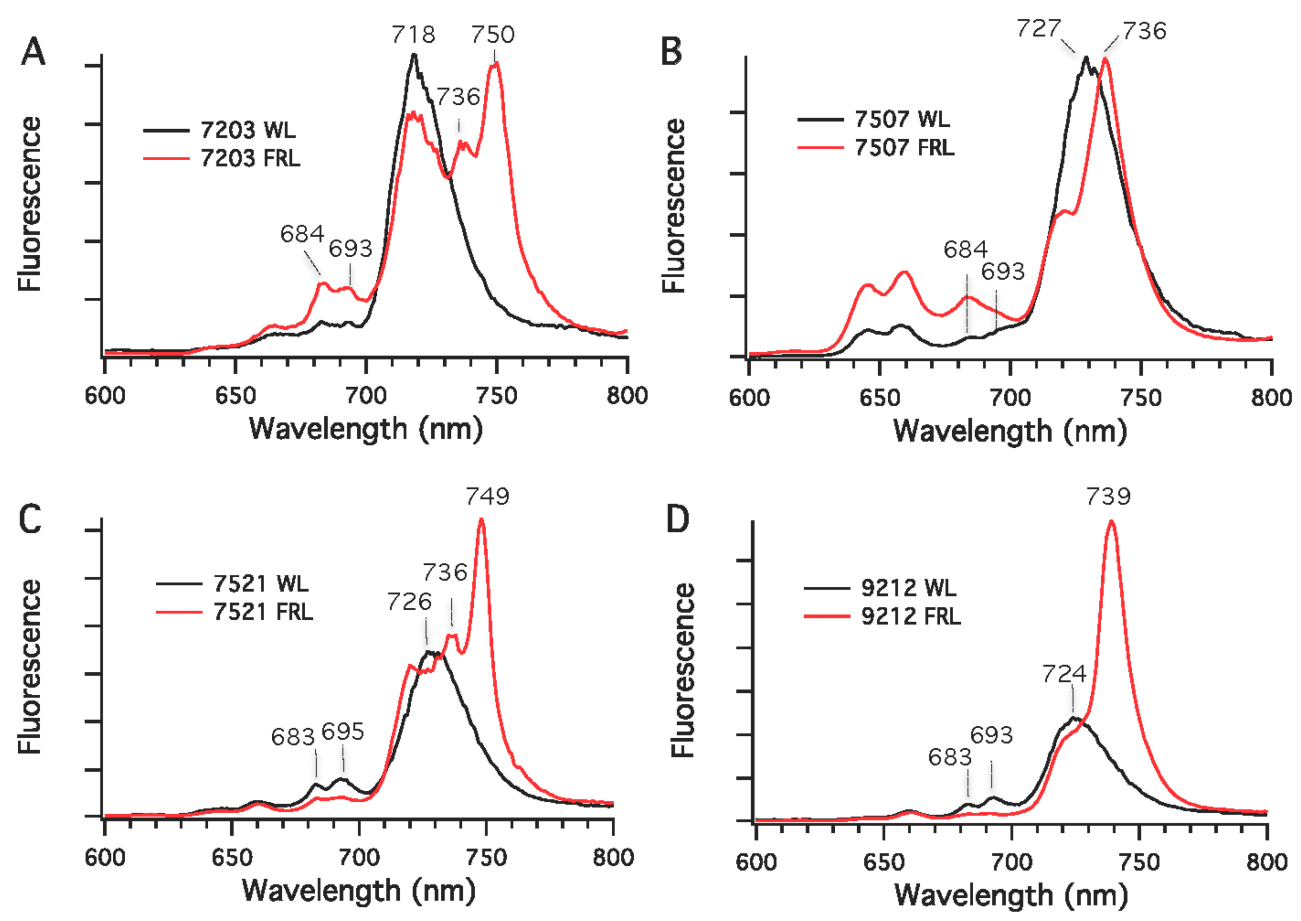
Low-temperature (77 K) fluorescence emission spectra for four cyanobacterial strains grown in white light (WL) or far-red light (FRL). (**A**) *Chr. thermalis* PCC 7203; (**B**) *Calothrix* sp. PCC 7507; (**C**) *F. thermalis* PCC 7521; (**D**) *Chlorogloeopsis* sp. PCC 9212. The excitation wavelength was 440 nm, which predominantly excites Chls.

Chls in cyanobacteria are mostly associated with PS I and PS II under nutrient-replete and moderate irradiance conditions. Low-temperature fluorescence emission spectroscopy was used to detect differences in the synthesis of photosynthetic complexes in cells grown in WL (or RL) and FRL conditions. As shown in Figure 6, the fluorescence emission maxima for PS I complexes in whole cells grown in WL had emission maxima at 720 nm for *Chr. thermalis* PCC 7203, 730 nm for *Calothrix* sp. PCC 7507 and *F. thermalis* PCC 7521, and 725 nm for *Chlorogloeopsis* sp. PCC 9212. As also observed in *Leptolyngbya* JSC-1 [28] and *Synechococcus* sp. PCC 7335 (Figure 2B), the amplitudes of the emission peaks from PS II at 683 and 695 nm in cells grown in WL were much smaller than that for PS I, which is expected because PS I binds the majority of the Chls in cyanobacteria [34]. The fluorescence emission spectra for cells grown in FRL had new, red-shifted emission peaks compared to cells grown in WL. For *Calothrix* sp. PCC 7507 and *Chlorogloeopsis* sp. PCC 9212, a major new emission peak occurred at 736 nm for the PS I complexes of these two strains. For *Chr. thermalis* PCC 7203 and *Chlorogloeopsis* sp. PCC 9212, two emission peaks with maxima at 736 and 750 nm were observed for PS I in cells grown in FRL. In these four strains, much lower fluorescence emission was observed between 680 and 700 nm for cells grown in FRL than for cells grown in WL. This observation implies that the PS II complexes of cells grown in FRL contain Chl *d*, Chl *f*, or both minor Chls (also see [28]). Thus, the five strains tested here can grow photoautotrophically in FRL, have enhanced absorption between 700 and 800 nm when grown in FRL, and synthesize Chls *d* and *f* in addition to Chl *a* when grown in FRL.

**Figure 7:**
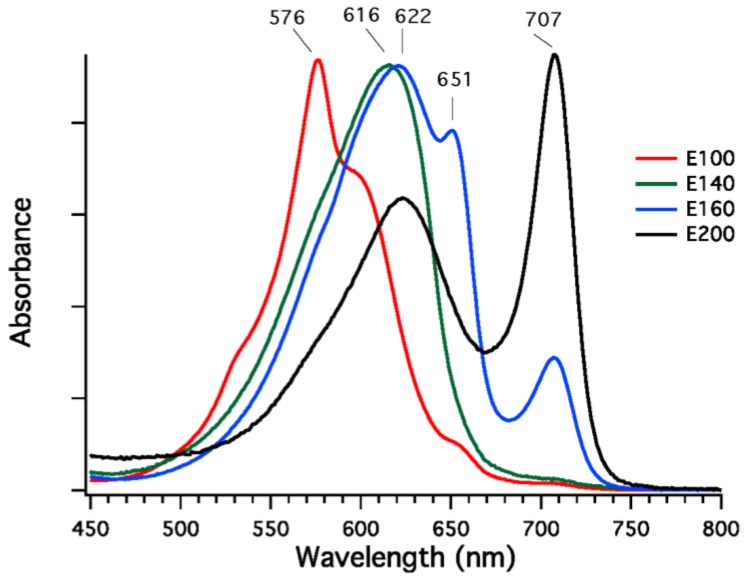
Absorption spectra of fractions from diethylaminoethyl (DEAE)-Sepharose chromatography of dissociated PBS isolated from *F. thermalis* PCC 7521 cells grown in far-red light (FRL). The buffer was 50 mM Tris-HCl at pH 7.0, and four fractions were eluted with 100, 140, 160, and 200 mM NaCl. Fraction E100 eluted with 100 mM NaCl (red line) has an absorption maximum at 576 nm and is highly enriched in phycoerythrocyanin [32]. Fraction E140 eluted with 140 mM NaCl (green line) has an absorption maximum at 616 nm and is highly enriched in phycocyanin. Fraction E160 eluted at 160 mM (blue line) has absorption maxima at 622, 651, and 707 nm. It is presently unclear whether this fraction is a mixture of proteins or whether this represents a component of the core substructure. Finally, fraction E-200 eluted with 200 mM NaCl (black line) has absorption maxima at 622 and 707 nm. This fraction resembles the spectrum of allophycocyanin-B [35] except that the long-wavelength absorption maximum is red-shifted by ~36 nm. See main text for additional details.

Phycobilisomes were isolated from *F. thermalis* PCC 7521 cells that had been grown in FRL, and fractions enriched in specific phycobiliproteins were isolated from dissociated phycobilisomes by DEAE-Sepharose chromatography at pH 7.0. Four fractions eluting at different sodium chloride concentrations (100 to 200 mM NaCl) were obtained, and the absorption spectra of these fractions are shown in Figure 7. The fractions eluting at 100 mM and 140 mM NaCl were highly enriched in phycoerythrocyanin (576 nm) and phycocyanin (616 nm), respectively [32]. The fraction eluted at 160 mM NaCl had absorption maxima at 620, 650, and 710 nm, and this fraction may either be a mixture of proteins or could be representative of allophycocyanin-like proteins derived from the core substructure. The fraction eluted with 200 mM NaCl is unlike any reported phycobiliprotein, although similar fractions have been obtained from *Leptolyngbya* JSC-1 cells grown in FRL [28]. This fraction had a broad absorption band with a maximal absorption at 622 nm and a very narrow and more intense absorption band with maximal absorption at 707 nm; the shape of the spectrum resembles that of allophycocyanin-B, which has maxima at 618 nm and 671 nm [35], except that the long-wavelength absorption band is red-shifted by ~36 nm. Very similar fractions were also observed for phycobiliproteins isolated from cells of *Synechococcus* sp. PCC 7335 grown in FRL, but no phycobiliproteins with absorption above 700 nm are present when cells of this strain are grown in WL or RL (see spectra in Figure 2A). These data show that, as previously observed for *Leptolyngbya* JSC-1 [28], phycobiliproteins comprising the core substructure are replaced and phycobilisomes remodeled in cells of *F. thermalis* PCC 7521 and *Synechococcus* sp. PCC 7335 undergoing FaRLiP.

## 3. Discussion

The results reported here show that five additional cyanobacterial strains (*Synechococcus* sp. PCC 7335, *Chr. thermalis* PCC 7203, *Calothrix* sp. PCC 7507, *F. thermalis* PCC 7521, and *Chlorogloeopsis* sp. PCC 9212), which have photosynthetic gene clusters similar to that in *Leptolyngbya* JSC-1 (see Figure 1), can perform FaRLiP and thereby grow continuously and photoautotrophically in FRL. Airs *et al.* [36] recently reported that the soil cyanobacterium, *Chlorogloeopsis fritschii* PCC 6912, a strain that seems to be very similar to *Chlorogloeopsis* sp. PCC 9212 studied here, can also grow in FRL and produces Chls *d* and *f* when it does so. *Chl. fritschii* has a photosynthetic gene cluster that is identical to that in *Chlorogloeopsis* sp. PCC 9212 (Figure 1). Considering that two of the remaining, untested strains with similar gene clusters are *Fischerella* spp. strains closely related to *F. thermalis* PCC 7521 [28], we conclude that the presence of such gene clusters can confidently be considered to be diagnostic of the capacity to perform FaRLiP. Furthermore, based upon the results obtained here, one can further predict that similar gene clusters will probably be present in *Halomicronema hongdechloris*, the first strain shown to synthesize Chl *f* [37,38,39] and *Aphanocapsa* sp. strain KC1 from Lake Biwa in Japan, which also synthesizes Chl *f* [40]. These Section 3 and Section 1 strains, respectively, increase the diversity of environments where FaRLiP has been detected. *H. hongdechloris* is a filamentous, non-heterocystous cyanobacterium closely related to *Leptolyngbya* spp.; this strain was isolated from a stromatolite from Shark Bay, Western Australia [38]. *Aphanocapsa* sp. strain KC1 is a unicellular cyanobacterium that was isolated from a bloom in a freshwater lake [40].

It is striking that exactly the same set of 17 photosynthesis-related genes, as well as the genes encoding the RfpA photoreceptor; the DNA-binding, signal receiver/response regulator, RfpB; and the signal receiver protein RfpC, occur in all 13 photosynthetic gene clusters in organisms likely to be capable of FaRLiP. The core genes for the photosynthetic complexes themselves appear to have arisen by many independent gene duplication events, which seemingly produced proteins that are optimized to perform light harvesting and energy conversion in FRL by binding Chls *a*, *d*, and *f*. These genes then somehow became clustered into a few operons, which came under the control of the knotless red/far-red phytochrome, RfpA, to produce a regulon that could lead to acclimation to and growth in FRL. The sporadic occurrence of this regulon and the associated gene clusters in a highly diverse group of cyanobacteria strongly implies that most of these organisms acquired this entire gene cluster by horizontal gene transfer. Evidence for horizontal transfer of an even larger photosynthesis gene cluster from a purple bacterium to a member of the phylum *Gemmatimonadetes* has recently been reported [3]. This evidence for horizontal gene transfer of photosynthetic functions has important implications for the evolution of photosynthesis, because horizontal gene transfer is often invoked to explain incongruence between the phylogenies of chlorophototrophs based on housekeeping functions and the photosynthesis-related genes they possess [4]. Organisms that belong to Section 4 and Section 5 (*Calothrix* sp. PCC 7507, *Chlorogloeopsis* spp., *Fischerella* spp., and *Mastigocoleus testarum*; see Figure 1 and [28]) are a possible exception to acquisition by horizontal gene transfer. Although some *Fischerella* spp. strains that have been sequenced completely lack the FaRLiP gene cluster, the gene cluster may have been specifically lost in those lineages. Consistent with the notion that these Section 4 and Section 5 strains have acquired the gene cluster vertically, RfpB, which is the DNA-binding, signal receiver/response regulator, differs slightly in these strains (Supplemental Figure S1). In the strains of Section 1, Section 2 and Section 3–3, RfpB has signal-receiver domains at both the *N*- and *C*-termini (Supplemental Figure S2). However, in the FaRLiP strains of Section 4 and Section 5, the RfpB proteins are missing the *C*-terminal receiver domain, and as a result RfpB proteins in these strains are nearly 300 amino acids smaller than those in the other strains. However, because the *N*-terminal regions of all RfpB proteins are very similar (Supplemental Figure S2), it seems very likely that the *C*-terminal receiver domain was specifically deleted in an ancestor of Section 4 and Section 5 strains, which produced an ancestral form of RfpB that was then vertically transmitted within these strains (Supplemental Figures S1 and S2).

Because of the current importance of cultivation-independent DNA sequencing methods in microbial ecology, it was of interest to assess whether the capacity to perform FaRLiP could be recognized on the basis of DNA sequence signatures inherent in the genes of the cluster. Furthermore, it was of interest to examine some of the specific properties predicted from conserved features of the protein sequences encoded with the FaRLiP gene cluster. Previous analyses of RfpA sequences from FaRLiP strains showed that these red/far-red knotless phytochromes form a structurally and phylogenetically distinctive clade [28]. Thus, the presence of sequences related to RfpA, and the knowledge that these sequences are correlated with the ability to perform FaRLiP as shown in this study, means that any sequence belonging to this clade predicts the existence/presence of an organism with the capacity to perform FaRLiP.

Sequence comparisons and phylogenetic analyses of some other proteins encoded in the photosynthesis gene cluster were conducted to determine if those sequences also formed distinctive sequence clades. Supplemental Figure S3 shows a phylogenetic tree of PsaA and PsaB sequences derived from a diverse group of cyanobacteria, including all of the strains that were confirmed to perform FaRLiP in this study. The PsaA2 and PsaB2 sequences, which are associated with FRL [28], form distinctive clades within the larger PsaA and PsaB sequence families. In each case, the sequences associated with the FaRLiP-specific, photosynthesis gene cluster were divergent and easily distinguishable from paralogous PsaA1 and PsaB1 sequences expressed under other light conditions (WL, GL, RL) (Supplemental Figure S3) [28]. Thus, phylogenetic analyses of PsaA and/or PsaB sequences might be used to infer the presence of an organism capable of performing FaRLiP in a metagenomic analysis.

A previous analysis of PsbA sequences in cyanobacteria identified so-called “rogue” and “super-rogue” PsbA sequences, which are highly divergent from “typical” PsbA sequences found in the PS II complexes of cyanobacteria [41]. The photosynthetic gene clusters in FaRLiP organisms (see Figure 1) contain two *psbA* genes, which are designated *psbA3* and *psbA4* in *Leptolyngbya* JSC-1 [28]. Transcriptional analyses showed that both of these *psbA* genes are transcribed in *Leptolyngbya* JSC-1 cells grown in FRL, and proteomic analyses showed that both of these polypeptides occur in fractions containing PS II complexes [28]. This could mean that two different types of PS II complexes are produced, one with PsbA3 and one with PsbA4. However, because PS II forms dimeric complexes, it could also mean that the PS II “dimers” are actually heterodimers in cells grown in FRL. PsbA3 is similar in sequence to typical PsbA sequences and has nearly all of the very highly conserved, functionally important residues, associated with cofactor ligation in these proteins (Supplemental Figure S4). However, PsbA4 is a “super-rogue” PsbA. PsbA4 is highly divergent from other PsbA sequences and lacks most of the residues believed to be functionally important, especially at the *C*-terminus where ligands to the tetra-Mn-containing, oxygen-evolving complex occur (Supplemental Figure S4; also see [41]). An intriguing possibility is that the PsbA3 and PsbA4 sequences combine to form a PS II heterodimer that would only have one functional electron transfer chain. Compared to the “homodimeric” PS II complexes produced in WL, which would have a functional electron transport chain in each PS II monomer, inclusion of a PsbA4 subunit that is inactive in electron transport would effectively double the number of antenna Chls associated with the functional P680 trap that would presumably be located in the PS II monomer containing PsbA3. While the functional role of super-rogue PsbA subunits is presently unclear, phylogenetic analyses that identify super-rogue PsbA sequences similar to PsbA4 could be used to demonstrate the presence of organisms performing FaRLiP. It should additionally be noted that some FaRLiP strains also have sequences belonging to the “rogue” PsbA clade (Supplemental Figure S5), the function of which is currently unknown [41]. Finally, it should also be noted that *Gloeobacter kilaueensis* has a highly divergent *psbA* gene, which encodes a protein with some functional similarities to super-rogue PsbA subunits [42]. However, preliminary phylogenetic analyses suggest that this protein is not a member of the super-rogue clade (data not shown). Although this PsbA protein has tyrosine Z and ligands to Chls, it lacks essential amino acid residues for binding the tetra-Mn cluster, lacks ligands to the non-heme Fe, but probably arose independently from the super-rogue PsbA4-like sequences.

We next examined the sequences for ApcE, the PBS core membrane linker phycobiliprotein. ApcE has an *N*-terminal, phycocyanobilin-binding domain that contains an inserted “loop” domain that is thought to play an important role in PBS attachment to PS II [15,17]. The chromophores bound to ApcE are believed to direct energy to PS II, while those associated with ApcD direct energy to PS I [15,16,17]. This *N*-terminal domain of ApcE, which is structurally related to other phycobiliproteins such as allophycocyanin (Supplemental Figure S6), contains a conserved cysteine residue to which phycocyanobilin is covalently attached. The binding pocket for this phycocyanobilin is found in a helix-turn-helix sequence between residues 180 and 230 in the alignment shown in Supplemental Figure S6. The conserved, chromophore binding cysteine residue in ApcE/ApcE1 proteins occurs at position 223 in this alignment. This attachment position has migrated from the *N*-terminus of the first helix (residue 183) to the *C*-terminus of subsequent helix (residue 223) in ApcE/ApcE1. Strikingly, there are no cysteines at all in the phycobiliprotein domains of ApcE2, although the binding pocket residues are well conserved (Supplemental Figure S6). These observations imply that phycocyanobilin is bound non-covalently to ApcE2; based on results from mutagenesis studies, this would red-shift the absorption and fluorescence emission maxima of the phycocyanobilin chromophore by ~40 nm [28,43,44].

ApcE proteins also contain two, three, or four *C*-terminal REP domains, which are linker domains that provide the scaffolding that determines the substructure organization of the PBS core. For example, ApcE1 of *Leptolyngbya* JSC-1 has four repeated linker (REP) domains, which should direct the assembly of a so-called pentacylindrical PBS core [15]. In contrast, the ApcE2 protein produced in FRL only has two REP domains and thus should direct the assembly of PBS with bicylindrical cores [28]. Among organisms that do not perform FaRLiP, only four organisms (*Synechococcus* sp. PCC 6301, 7942, and 7336 and *Trichodesmium erythraeum* IMS101) synthesize ApcE proteins with only two REP domains. Phylogenetically, however, these proteins belong to the ApcE1 sequence clade that includes proteins with 2, 3 and 4 REP domains (Supplemental Figure S7). Phylogenetic analyses also show that ApcE2 forms a distinctive clade that is easily distinguishable from the ApcE1 sequences found in strains that can perform FaRLiP, even when one includes the structurally similar proteins with only 2 REP domains. As described above, all thirteen of the ApcE2 sequences lack the conserved cysteine residue required for covalent attachment of phycocyanobilin to the proteins. These analyses indicate that ApcE sequences can probably be used to identify strains capable of performing FaRLiP.

We also analyzed the ApcA, ApcB, ApcD, and ApcF sequences in organisms capable of FaRLiP. Supplemental Figure S8 shows a neighbor-joining phylogenetic tree of the sequences for strains with FaRLiP gene clusters and for a few other selected strains. The alignments used in making the phylogenetic tree are shown in Supplemental Figures S9 and S10 for the ApcA/ApcD sequence and the ApcB/ApcF sequence families, respectively. As shown in Figure 7, when FaRLiP strains are grown in FRL, cells synthesize unusual phycobiliproteins with absorption at wavelengths longer than 700 nm. This unusual property led us to postulate that the sequences for the phycobiliproteins subunits encoded within the FaRLiP photosynthetic gene cluster would form distinctive clades not found in other cyanobacteria, and this is the case (Supplemental Figure S8). The ApcA and ApcD sequences formed 7 clades, while the ApcB and ApcF sequences formed 5 clades (Supplemental Figure S8). Most of the expansion of the alpha-type sequence family was produced by gene duplications involving ApcD rather than ApcA. The ApcD-related sequences form five clades, while the ApcA sequences form only two clades, one of which (ApcA3) includes sequences that are only found in a few cyanobacteria. ApcD1 encodes the alpha subunit of allophycocyanin-B, which is one of the “terminal emitters” in the core substructure of phycobilisomes and is associated with energy transfer to PS I [15,16,17]. Because ApcD1 already had long-wavelength absorption (λ_max_ = 671 nm) [15,35], it is perhaps not surprising that this protein family expanded in organisms that can grow in far-red light.

In FaRLiP organisms the ApcA1 and ApcD1 proteins are used to form phycobilisome cores in WL, GL, and RL, and the phycobilisomes produced in FRL contain ApcB2 and ApcA2, ApcD2, and ApcD3 [28]. Although the *apcA2* gene is upstream from and apparently cotranscribed with *apcB2* in all FaRLiP gene clusters (Figure 1), ApcA2 is actually more closely related to ApcD1 than to ApcA1, and ApcA2 should probably be renamed ApcD5 for this reason. As can be inferred from the phylogenetic analysis shown in Supplemental Figure S8, ApcA1 is replaced by ApcD-like sequences (ApcA2/ApcD5, ApcD2, and ApcD3) in cells growing in FRL. This helps to explain the unusual, long-wavelength-absorbing phycobiliproteins found in phycobilisomes produced in far-red light (see Figure 7). Interestingly, two of the ApcD3 sequences, those from *Synechococcus* sp. PCC 7335 and *Chr. thermalis* 7203, lack the otherwise universally conserved cysteine residue (replaced by alanine) at position 81, which is the attachment site for phycocyanobilin (Supplemental Figure S9). As discussed for ApcE2, these two proteins should bind phycocyanobilin non-covalently, which would retain one additional conjugated double bond relative to the covalently bound form and which would red-shift the absorption spectrum ~40 nm. Several of the paralogous ApcA and ApcD subunits synthesized in FRL have additional cysteine residues (Supplemental Figure S9), which raise the intriguing possibility that some of these subunits may bind additional chromophores or be subject to redox regulation. Finally, three FaRLiP strains have proteins belonging to the ApcD4 clade, which are also found in three cyanobacteria that do not perform FaRLiP (Supplemental Figure S8). The *apcD4* gene is colocalized with and apparently cotranscribed with *apcB3*, but the conditions under which these genes are expressed and the function of their products are not known.

The situation for the ApcB and ApcF sequences is similar to that for ApcA and ApcD. Three clades of ApcB sequences, denoted ApcB1, ApcB2, and ApcB3, were identified, and only a few strains have sequences belonging to the latter clade, the genes for which are colocalized with *apcD4* as noted above. ApcB2 replaces ApcB1 when cells are grown in FRL [28]. All ApcB2 sequences have an additional cysteine residue at position 77, but the function of this residue is presently unknown. Most cyanobacteria have a single copy of the *apcF* gene, which suggests that ApcF can associate functionally with either ApcE1 or ApcE2 in the cores of phycobilisomes [15]. However, two strains, *Chr. thermalis* PCC 7203 and *Oscillatoria* sp. JSC-12, have two copies of ApcF, which form a separate clade (Supplemental Figure S10). The conserved cysteine for phycocyanobilin binding is replaced by tyrosine in ApcF2, which strongly implies that this protein carries a non-covalently bound phycocyanobilin residue as discussed above for ApcE2 and ApcD3. The *apcF2* gene is a part of the FaRLiP gene cluster in *Oscillatoria* sp. JSC-12 [28], which strongly implies that ApcF2 is a specific binding partner for ApcE2 when cells are grown in FRL.

## 4. Conclusions

We have shown in this study that the capacity to perform FaRLiP is positively correlated with the presence of photosynthetic gene clusters similar to the one originally identified and characterized in *Leptolyngbya* JSC-1 [28]. Each of these gene clusters contains alternative genes for core components of PS I, PS II, and phycobilisomes (*apcA2*/*B2*/*D2*/*E2*/*D3*, *psbA3*/*D3*/*C2*/*B2*/*H2*/*A4*, *psaA2*/*B2*/*L2*/*I2*/*F2*/*J2*) as well as a red/far-red knotless phytochrome and two response regulators. Organisms capable of FaRLiP occur in all five taxonomic sections of the phylum *Cyanobacteria*, and most of these strains are derived from terrestrial habitats. Many of the genes in the FaRLiP photosynthetic gene cluster are sufficiently divergent from their paralogs so that these sequences can confidently be identified and used to predict the occurrence of FaRLiP in metagenomic surveys. Using targeted blastP and blastX searches and phylogenetic analysis, we failed to find any evidence for FaRLiP in the extremely large Global Ocean Survey database [45], but we identified sequences indicating the occurrence of FaRLiP in a metagenomic dataset for a cyanobacterial streamer community in White Creek in Yellowstone National Park, WY [46]. Current evidence suggests that FaRLiP will most likely be associated with light niches where far-red light predominates due to strong filtering by Chl *a* or because of far-red light enrichment due to scattering (e.g., by soil particles). FaRLiP is likely to play an important role in terrestrial photosynthesis by enhancing the photosynthetic properties of the organisms living in such environments.

## 5. Materials and Methods

### 5.1. Strains and Growth Conditions

The cyanobacterial strains used in this study were obtained from the Pasteur Culture Collection (http://www.pasteur.fr/pcc_cyanobacteria) [47,48]. *Synechococcus* sp. PCC 7335 was isolated from a snail shell in an intertidal zone near Puerto Penasco, Mexico. *Chrooococcidiopsis thermalis* PCC 7203 was isolated from soil near Greifswald, Germany. *Calothrix* sp. PCC 7507 was originally isolated from a sphagnum bog near Kastanienbaum, Switzerland. *Fischerella* sp. PCC 7521 strain was originally isolated from a hot spring near Mammoth Sinkhole II in Yellowstone National Park, USA. *Chlorogloeopsis* sp. PCC 9212 was isolated from a thermal feature near Ourense, Spain.

*Synechococcus* sp. PCC 7335 was cultured in ASN III growth medium [48] to which vitamin B_12_ (10 μg·mL^−1^, final concentration) and Tris-HCl, pH 8.0 (10 g·L^−1^, final concentration) were added. In some experiments, *Synechococcus* sp. PCC 7335 was grown mixotrophically by adding 1 mM fructose to the medium. The other four strains were grown in the B-HEPES growth medium [49], a modified BG11 medium containing 1.1 g·L^−1^ HEPES (final concentration) with the pH adjusted to 8.0 with 2.0 M·KOH. Warm white fluorescent bulbs provided continuous illumination at ~250 µmol photons m^−2^·s^−1^ (WL), and liquid cultures were sparged with 1% (v/v) CO_2_ in air. *Synechococcus* sp. PCC 7335 was grown at a lower WL intensity of ~100 µmol photons m^−2^·s^−1^. Red light (RL) was provided by light-emitting diodes (LEDs) with emission centered at 645 nm (~50 µmol photons m^−2^·s^−1^) (Marubeni, Santa Clara, CA, USA). Far-red light was provided by LEDs (Marubeni, Santa Clara, CA, USA) with emission centered at 720 nm (15–18 µmol photons m^−2^·s^−1^). Plastic filters, for which the transmittance properties have been described previously [28], were sometimes used to produce green light (GL), red light (RL), and FRL. Growth of cells was monitored turbidometrically at 730 nm by using a GENESYS 10 spectrophotometer (ThermoSpectronic, Rochester, NY, USA).

### 5.2. Total Pigment Measurements and Absorption and Fluorescence Spectroscopy

The Chl and carotenoid contents of cells were measured from pigments extracted with 100% methanol. Spectroscopic measurements were performed with a GENESYS 10 UV–Vis spectrophotometer (ThermoSpectronic, Rochester, NY, USA). The Chl *a* concentration was determined on the basis of equivalent cell concentrations as determined by equal OD_730 nm_ values as described [50]. To measure the absorption spectra of whole cells, cells were harvested and resuspended in 10% (w/v) sucrose prepared with 50 mM HEPES buffer, pH = 7.0. Homogenization with a Teflon/glass homogenizer was used to achieve a more homogenous resuspension of the filamentous cells of some of the cyanobacterial strains employed in this study. Absorption spectra of cell cultures were measured with a UV–Vis–NIR Cary™ 14 spectrophotometer that has been modified for computerized data collection and analysis by On-Line Instrument Systems, Inc. (Bogart, GA, USA).

Fluorescence emission spectra at low temperature (77 K) were measured using an SLM Model 8000C spectrofluorometer that has been modified for computerized solid-state operation by On-line Instrument System In., (Bogart, GA, USA) as described [50]. Cells were collected and resuspended in 50 mM HEPES/NaOH, pH = 7.0 buffer containing 60% (v/v) glycerol through gentle homogenization using a homogenizer. After loading samples into the measuring tubes, cells were quickly frozen in liquid nitrogen. To measure the fluorescence emission from Chl-protein complexes, the excitation wavelength was 440 nm, which selectively excites Chls.

### 5.3. Pigment Extraction and HPLC Analysis

For pigment extraction and HPLC analyses, cyanobacterial cells were harvested and washed once in 50 mM HEPES/NaOH buffer, pH = 7.0 by gentle centrifugation, resuspension, and subsequent centrifugation. The following methods have been described previously [28,51], and were conducted under the dim light. Pigments were extracted from the cell pellet by sonication in acetone:methanol (7:2, v/v). After centrifugation to remove protein and other insoluble cell debris, pigment solutions were filtered through a 0.2-µm polytetrafluoroethylene membrane syringe filters. Pigments were analyzed by reversed-phase HPLC on a 25 cm × 4.6 mm analytical Discovery C18 column (Supelco, Bellefonte, PA, USA) using an Agilent Model 1100 HPLC system equipped with a model G1315B diode array detector. The solvent system and elution conditions were identical to those previously described by Gan *et al.* [28].

### 5.4. Phycobilisome Isolation and Ion-Exchange Chromatography

Phycobilisomes were isolated as previously described [28], and the fraction collected from the sucrose gradients was dialyzed against 50 mM Tris-HCl buffer at pH 7.0. The resulting dissociated phycobiliproteins were applied to a DEAE-Sepharose column (1.0 × 12 cm). After the bound proteins were washed with a few column volumes of starting buffer, fractions were eluted with increasing step concentrations of NaCl (100, 140, 160, and 200 mM NaCl).

### 5.5. Bioinformatics and Phylogenetic Analyses

Amino acid sequence data for homologs of the polypeptides PsaA, PsaB, PsbA, ApcA, ApcB, ApcD, ApcE, ApcF, and RfpB were obtained from the Integrated Microbial Genomes Database of the DOE Joint Genome Institute (IMG-JGI) at http://img.jgi.doe.gov/ and from CyanoBase at http://www.kazusa.or.jp/cyano/. Analyses of protein sequences were conducted using MEGA6 [52]. Sequence alignments and phylogenetic analysis were performed using the MUSCLE [53] module in MEGA6, and neighbor-joining phylogenetic trees were produced and evaluated by the bootstrap method with MEGA6.

## Supplementary Materials

Supplementary materials can be accessed at: http://www.mdpi.com/2075-1729/5/1/4/s1.
