# Supplementary material for: Occurrence of Far-Red Light Photoacclimation (FaRLiP) in Diverse Cyanobacteria
Source: Life (Basel). 2014 Dec 29;5(1):4–24. doi: 10.3390/life5010004 (PMC4390838; doi:10.3390/life5010004)
Supplement: Supplementary file 1 [file life-05-00004-s001.pdf]

Supplementary Material

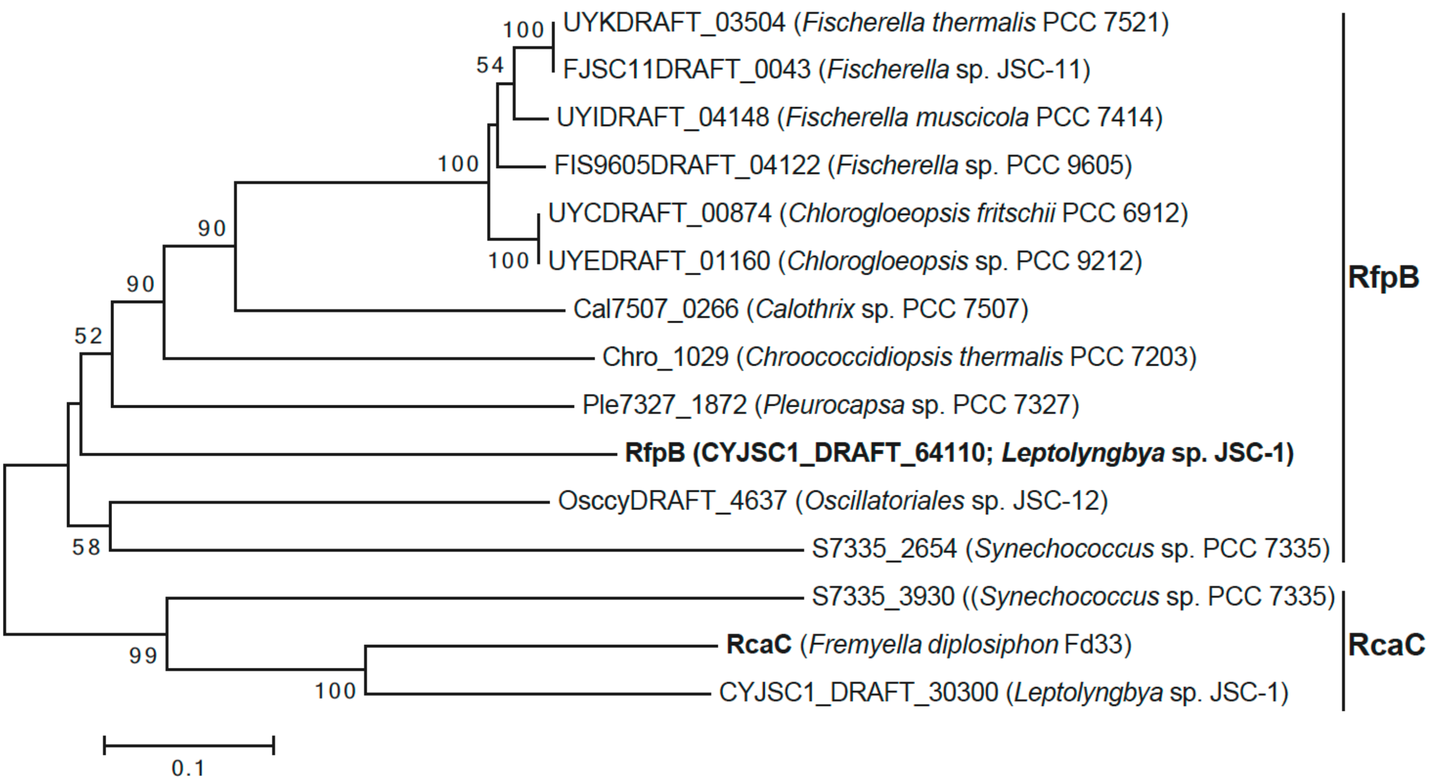

Figure S1. Phylogenetic tree for RfpB sequences from strains with photosynthetic gene clusters similar to that in *Leptolyngbya* JSC-1. Sequences were aligned using the the MUSCLE module implemented in MEGA6, and a neighbor-joining tree was built from the resulting sequence alignment. Numbers at the nodes indicate the bootstrap support based upon 100 resamplings. A few RcaC sequences, the signal receiver/response regulator protein for CCA, were included as outgroup.

```
                           10         20         30         40         50         60         70         80         90        100
S7335_2654         MRILLVEDDESVAKILHKVLTDEHYIVDVAYTGHIGWQLVSSFNYDLIILDVLLPELDGIKFCQQLRAQEYTLPVLFVTALDDSSEKIAGLNAGADDYIT
Chro_1029          MRILLIEDDECVAKALKNVLQQQNYVVDTATDGQTGWDLIQTFTYDLILLDVILPKLDGIKLCQRLRDRKYQTPVLLITAQDSSDRKVMGLDAGADDYLV
Ple7327_1872       MRILVVEDDKSIAETLERVLGEQHYVVDAATDGEMGWELVEVFAYDLIVLDVMLPKLNGIEFCQRLRSRGDRTPILLLTAKSSSTDKTTGLNAGADDYVV
CYJSC1_DRAFT_64110 MKVLLVEDDECTAKTLETALARENYAIDVAGDGELGWRLIEAFAYDLILLDVILPKLDGISLCRQIRQHNYKTPVLLLTAQNSSTDRIAGLDAGADDYVV
OsccyDRAFT_4637    MRILLVEDDESISRALEAILTEHHYVVDVAADGQIGWECVEAFTYDLIVLDVLLPRLDGIQFCQQLRAQDCQIPVLLLTAQSSSRDRIIGLDAGADDYMV
Cal7507_0266       MRILLVEDDECIAKTLERVLGNQNYVVDIAADGLLGWELVEAFSYDLILLDIILPKLDGIKLCQRLRSHNYQTPILLLTAQNSSTHKVMGLDAGADDYVV
UYEDRAFT_01160     MRLLLVEDDECIADTLEKILGNHHYVVDVANDGELGWELVEAFNYDLILLDVMLPKLDGIQLCQRLRSHNYQAPVLLLTAQNSSTHKVMGLDAGADDYLV
UYCDRAFT_00874     MRLLLVEDDECIADTLEKILGNHHYVVDVANDGELGWELVEAFNYDLILLDVMLPKLDGIQLCQRLRSHNYQAPVLLLTAQNSSTHKVMGLDAGADDYLV
FIS9605DRAFT_04122 MRLLLVEDDECIANTLENILGNHHYVVDVANDGELGWELVEAFNYDLILLDVMLPKLDGIQLCQKLRSHNYQAPVLLLTAQNSSTHKVMGLDAGADDYLV
FJSC11DRAFT_0043   MRLLLVEDDECIANTLENILGNQHYVVDVANDGELGWELVEAFKYDLILLDVMLPKLDGIQLCQRLRSHNYQAPVLLLTAQNSSTHKVMGLDAGADDYLV
UYKDRAFT_04148     MRLLLVEDDECIANTLENILGNQHYVVDVANDGELGWELVEAFKYDLILLDVMLPKLDGIQLCQRLRSHNYQAPVLLLTAQNSSTHKVMGLDAGADDYLV
UYKDRAFT_03504     MRLLLVEDDECIANTLENILGNQHYVVDVANDGELGWELVEAFKYDLILLDVMLPKLDGIQLCQRLRSHNYQAPVLLLTAQNSSTHKVMGLDAGADDYLV

                          110        120        130        140        150        160        170        180        190        200
S7335_2654         KPFELEELLARVRALLRRGKTSVVLTTLEWEGLRLEVNSRELTYRNNKLSLTPKEHAMLELFLRNPAQAFSREAIIDNLWSYSDAPGEETVTSHMKGLRR
Chro_1029          KPFDMPELLARIRVLLRR-QSKPILQALEWGDLRLQCGTCEVTYGDRVLHLTPNEYRLLELFLRHPQLVLSRGEILDRVWAVPEAPKEDTVTAHIKGLRQ
Ple7327_1872       KPFDLPELLARIRVLLRR-KNSLIPPVLEWGDLRLDPNTCEASYGSQPLRLTPKEYRLLELFLRNPQQVLTRNTILENLWSVEETPSEDTITAHIKSLRQ
CYJSC1_DRAFT_64110 KPFELPELLARVRVLLRR-GSSFVLPVLEWDHLRLDLAACQVTYGDQLLQLTPKEYRMLELFLRHPQRVFARSSILEHLWGSEEMPGEDTVTSHIKGLRQ
OsccyDRAFT_4637    KPFEPQELLARLRVLLRR-KGSARPAVLEWMALRLDPSLCEVTYDGHLVSLTPKEYRLLELFLRHPQRIFSRSVILDHLWSCEEAPGEDTVTVHIKGLRQ
Cal7507_0266       KPFELSELLARIRVLLRR-RNSPILSVMKWEKLRINSGSCEVTYGCHGLNLTPKEYRLLELFLRNEHIVFSRSEILDHLWSIEEAPKEDTVTAHIKGLRQ
UYEDRAFT_01160     KPFDMSELLARIRVLLRR-RNSPIQTVLEWENLRLDPGKCEVLYNNHLLNLTPKEYRLLELFLRNGHQVFSRSDILEHLWATEEAPQEDTVTAHIKGLRQ
UYCDRAFT_00874     KPFDMSELLARIRVLLRR-RNSPIQTVLEWENLRLDPGKCEVLYNNHLLNLTPKEYRLLELFLRNGHQVFSRSDILEHLWATEEAPQEDTVTAHIKGLRQ
FIS9605DRAFT_04122 KPFDMSELLARIRVLLRR-RHSPIQTVLEWENLRLDPGKCEVTYNSHLLNLTPKEYRLLELFLRNGHQVFSRGDILEHLWSIEEAPQEDTVTAHIKGLRQ
FJSC11DRAFT_0043   KPFDMSELLARIRVLLRR-RNSTLQTVLEWENLRLDPGKCEVVYNNHLLNLTPKEYRLLELFLRNGHQVFSRGDILEHLWSTEEAPQEDTVTAHIKGLRQ
UYKDRAFT_04148     KPFDMSELLARIRVLLRR-RNSTLQTVLEWENLRLDPGKCEVVYNNHLLNLTPKEYRLLELFLRNGHQVFSRSDILEHLWATEEAPQEDTVTAHIKGLRQ
UYKDRAFT_03504     KPFDMSELLARIRVLLRR-RNSTLQTVLEWENLRLDPGKCEVVYNNHLLNLTPKEYRLLELFLRNGHQVFSRGDILEHLWSTEEAPQEDTVTAHIKGLRQ

                          210        220        230        240        250        260        270        280        290        300
S7335_2654         KLRTVGAPADLIETVYGVGYRLKPIETEVKPIETEELCSVDSRSA---------------------------------------------------TSDI
Chro_1029          KLKQVGAPADLIETVYGLGYRLKQPAPTTKKTKLRGASLPQSQDS-------------------------------------------------------
Ple7327_1872       KLKRAGSPPDSIATVYGIGYRLKSSAPSASEASCAPSATDKRAIS---------------------------------------------------SSIA
CYJSC1_DRAFT_64110 KLKQAGAPFNFIETVYGMGYRLRQSKS--EPSQSVPGNAQAAGQKQAPIILNGNGSNKNGSNKNGSNENGSNKNGSNKNGLNKNSLNQVDTAGNTNQSTD
OsccyDRAFT_4637    KLKRQGAPPDLIQTVYGQGYRLKQPSEFTASPPALPGWVRDGLVQ-------------------------------------------------------
Cal7507_0266       KLTQAGAPTDFIETVYGLGYRLKAFADEEKPSVASRQTKKSQSKR-------------------------------------------------------
UYEDRAFT_01160     KLKQAGAENDFIETVYGLGYRLKVFTSSQKMSQKTKENHRLNGAK---------------------------------------------------PSTK
UYCDRAFT_00874     KLKQAGAENDFIETVYGLGYRLKVFTSSQKMSQKTKENHRLNGAK---------------------------------------------------PSTK
FIS9605DRAFT_04122 KLKQAGAENNFIETVYGLGYRLKVPTSSQKLPQQTKENHRLNGVK---------------------------------------------------PSTK
FJSC11DRAFT_0043   KLKQAGAENNFIETVYGLGYRLKVPTSSKKLPQKTKENHRLNGVK---------------------------------------------------PSKK
UYKDRAFT_04148     KLKQAGAENDFIETVYGLGYRLKVFTSSQKMSQKTKENHRLNGAK---------------------------------------------------PSTK
UYKDRAFT_03504     KLKQAGAENNFIETVYGLGYRLKVPTSSKKLPQKTKENHRLNGVK---------------------------------------------------PSKK

                          310        320        330        340        350        360        370        380        390        400
S7335_2654         VKLRH--------------QHTQAALSALWKSVKSQQTNRLAILQQAFEQLQRGTLSKEMRESAYRSSHSLTGVLGIFGLETGSEIARQIQILLQGETPI
Chro_1029          -----------------IQQQTKAALAEVWEKIKYQSSDRVAVLEQATISLLDNKLTEELREKARQAAHKLAGALGIFGFAKGSRLAKHIEDIWQADVIL
Ple7327_1872       ISYSD-------ATRSRIAQQTKSGLKVVWEKFQGQTKARVTILEQATAALRDDALDEELRQQAKAAAHKLAGTLGVFGFPEGSRLAKEIEQVFSKKTIV
CYJSC1_DRAFT_64110 QALANPTQIQQDLETGRQKQQIQVGLQQLWSKYAEHNFNRLRLIEQAIEALQTGELSAALHHQAYVAAHQLAGAMGIFQRLHGSHLAAQLEQAFKSDQLT
OsccyDRAFT_4637    -------------------QKTRAGLADVWAKYKHLNCDRCLVLEQAGASMLVGTLTEELHQQACRTAHQLAGALGIFGLTDGSNLARRIEHLLQSSPLP
Cal7507_0266       ------------------KPQIREALAVVWERLKVHSSDRLEIIQQASLELQANNLSEELRKKAYHAAHKLGGALGIFGFAEGSRLAKHIEQILLSQIIL
UYEDRAFT_01160     QQAEDLFYEVQDLHNG--IQQTKAALTKLWEKIKDKSCDRIIILEQATAALLENNLGDELRQKAQQAAHKLAGALGIFGFSKGSRLAFEVEQMLRSQKKI
UYCDRAFT_00874     QQAEDLFYEVQDLHNG--IQQTKAALTKLWEKIKDKSCDRIIILEQATAALLENNLGDELRQKAQQAAHKLAGALGIFGFSKGSRLAFEVEQMLRSQKKI
FIS9605DRAFT_04122 QQTRDLCYETKDLQNG--IQQTKAALTKLWEKLKVKSCDRIIILEQATAALLENNLGDELRQQAQQAAHKLAGALGIFGFTKGSRLAFEVEQMLRSQMSI
FJSC11DRAFT_0043   QQIKDLFYEAQDLHDN--IQQTKAALTKLWEKLKVKNCDRIIILEQATEALLENNLGDELRQQAQQAAHKLTGALGIFGFTKGSRLAFEVEQMLRSHMNI
UYKDRAFT_04148     QQIRDLFYEAQDLHNN--IQQTKAALTKLWEKLKVKSCDRISILEQATAALLENNLGDELRQKAQQAAHKLAGALGIFGFIKGSRLAFEVEQMLRSHMNI
UYKDRAFT_03504     QQIKDLFYEAQDLHDN--IQQTKAALTKLWEKLKVKNCDRIIILEQATEALLENNLGDELRQQAQQAAHKLTGALGIFGFTKGSRLAFEVEQMLRSHMNI

                          410        420        430        440        450        460        470        480        490        500
S7335_2654         LADDQARLDALIRALQATIDE-AIEHRGRTAVEIPKVPLLVLVDPQLRMMPALVGMLRTEGFTVKISHHIESLVAMLSSSRLLDKKAGINQTDRTNQTDS
Chro_1029          DRSEALHLYEMVMALKREIQR-PSLSQLDRPLPRQQSIVMLAIDGDAGLAEQIVKLAATAGIGVKIAPNISAAKAAIAHLE-KFNRLS------------
Ple7327_1872       -PTRSSHLSKRVVALRQLLEE-SSASQIAEPIARHRSPVLLVISDDAKAARRLVELAVASKIRIRLVLSITAAKEALAH--------G------------
CYJSC1_DRAFT_64110 LREKHAALIAIIAELRQVLQAGAPLPEVGT------TSLLLVIDHDPTLLEQVRQ----SGQAIVLQPSDLAAVADSIHLA-KLAPSD------------
OsccyDRAFT_4637    TPNKAVQLLDLLNQLKEAIQS-PP-SHHNR------VPLLLVIEDDEELADQVFSLAMTYGIEMERLPSLLASHEHNELPEIDEAGMG------------
Cal7507_0266       DQSEALRLRHLVELLSGELQQ-PAFRELDK----------------------------------------------------------------------
UYEDRAFT_01160     NQNQILHLCNLVIALKQEIQQ-PAFAELEK----------------------------------------------------------------------
UYCDRAFT_00874     NQNQILHLCNLVIALKQEIQQ-PAFAELEK----------------------------------------------------------------------
FIS9605DRAFT_04122 NQSQVLHLYNLVIALKQEIQQ-PAFAELEK----------------------------------------------------------------------
FJSC11DRAFT_0043   NQNQVLHLYNLVIALKQEIQQ-PAFAELEK----------------------------------------------------------------------
UYKDRAFT_04148     NHNQVLHLYNLVIALKQEIQQ-PAFAELEK----------------------------------------------------------------------
UYKDRAFT_03504     NQNQVLHLYNLVIALKQEIQQ-PAFAELEK----------------------------------------------------------------------
```

Figure S2. *Cont.*

```
                          510       520       530       540       550       560       570       580       590       600
        S7335_2654 RTRSERTVPDVVLLGFSFADFEDDCESDLNDDLDRDLRSSQLDRLAKLVNEVPSLMLLICSGDGSLSSRVRAAQLGDYPFFYNPDVASVAKTIALLRSHP
         Chro_1029 -------PADVVILNLSLANTTAA----------------DLAILAELTNRTPPIPVLLCTRGDRLSDRIELSRLGVHAFLRQPFNPEEVLAAVTRLQQT
      Ple7327_1872 -------SLDVVLLVFSLTNAAKD----------------NLAFLANLINQTPPTPVLFLTSSDNLTVRIEAARLCDRAFFQKLQLPERGLATMNKVLKQ
CYJSC1_DRAFT_64110 -------QPATFVVNLCLADNLAA----------------HLAAFRQLTHQLPPTSILLFTDQADLNLRVQFTQAGVHAVLPKS-LVGQILSLAGQPAAQ
   OsccyDRAFT_4637 -------LGGEPVLLLNLTAGEEK----------------RLTLPATYSQQAAPPLVLFIANQNNLNTRVKAARLGAHVFLDKTELQSLVVQMTSNFSCQ
      Cal7507_0266 ---------------------------------------------------------ILCHY--------------------------------------
    UYEDRAFT_01160 ---------------------------------------------------------ILCHD--------------------------------------
    UYCDRAFT_00874 ---------------------------------------------------------ILCHD--------------------------------------
FIS9605DRAFT_04122 ---------------------------------------------------------ILCHD--------------------------------------
  FJSC11DRAFT_0043 ---------------------------------------------------------ILCHD--------------------------------------
    UYKDRAFT_04148 ---------------------------------------------------------ILCHD--------------------------------------
    UYKDRAFT_03504 ---------------------------------------------------------ILCHD--------------------------------------
                                                                                  .

                          610       620       630       640       650       660       670       680       690       700
        S7335_2654 LSAPR-----------------------KILAVDDDSQLLQTLKTRLALENYQIVTLDKPLEFWKTLQDENPDLLLLDISMPQFTGIELCQTVRRSPAWR
         Chro_1029 RSHPT-----------------------KILVVDDDPEILTLMQALLEPWGIQLNTLEEPSRFWAILEEFAPDMLVLDVEMPQFNGIDLCQIVRNEPRWH
      Ple7327_1872 IRRRV----------------------AKVMVVDDDPQMLYVMKTLLEIWGIKVTTLEEPSRFWETLAKFSPDLLILDVEMPIFNGIELCRVVRNDPYWC
CYJSC1_DRAFT_64110 PSIPW-----------------------QVLMVDDDPQILAAMQTWLSPWNIHLTALEHAKQLWQTLQTCDPDLLILDVEMPQINGIELCRAIRSEPRWQ
   OsccyDRAFT_4637 AACPTRPLAAQILEVVKQAKSHVCTTTAKVMVVDDDPQILAGIRALLEPWGLQLITLEEPRQFWQTLETFSPDLLLLDVQMPHFSGFELCRVVRNAPRWK
      Cal7507_0266 -------------------------------VVDDVGE
    UYEDRAFT_01160 -------------------------------VLDDA------------------------------------------IEQSPFKGVK
    UYCDRAFT_00874 -------------------------------VLDDA------------------------------------------IEQSPFKGVK
FIS9605DRAFT_04122 -------------------------------VLDDAIE
  FJSC11DRAFT_0043 -------------------------------VLDDAIE
    UYKDRAFT_04148 -------------------------------ILDDAIE
    UYKDRAFT_03504 -------------------------------VLDDAIE

                          710       720       730       740       750       760
        S7335_2654 HLPIVFFTAHAEPHLQQAAFKAGGDNLIEKSLSDAQLLAHLEHQMKRSYLQQVIATANSPTTVPP
         Chro_1029 KLPVLFLTAHTDDRTIQQVFTAGANDCLSKSILGTELVTQIFNRLGQMRLMQSIQ
      Ple7327_1872 GLPIVFVSVHTDEDTVNQILRAGGDDRISKSLIGPELVNRILNRLERVHTLRSLTNANNPQLR
CYJSC1_DRAFT_64110 QLPIVFLTVHRDSETLRQMLEVGANALINKPIGESSALLPILTQIKRAQVMRVMQ
   OsccyDRAFT_4637 DLPVLFLTMHLDTAKVQQAIAAGGDDYVSKATDTKELVTCILNRLEHTRLRRLLSSSTRSAYE
      Cal7507_0266
    UYEDRAFT_01160
    UYCDRAFT_00874
FIS9605DRAFT_04122
  FJSC11DRAFT_0043
    UYKDRAFT_04148
    UYKDRAFT_03504
```

Figure S2. Sequence alignment for RfpB sequences from various strains containing photosynthetic gene clusters similar to that in *Leptolyngbya* JSC-1. Sequences were aligned using the MUSCLE module implemented in MEGA6. The red lines indicate the CheY-like signal-receiver domains at the N-terminus (residues 1 to 120) and C-terminus (residues 630 to 735). The green line indicates the DNA-binding, winged-helix domain (residues 128 to 233). The blue line indicates the histidine-containing phosphotransferase (HPT) domain (residues ~300 to 430).

Figure S3. Phylogenetic tree of PsaA and PsaB sequences from diverse cyanobacteria. Sequences were aligned using the MUSCLE module implemented in MEGA6, and a neighbor-joining tree was built from the resulting sequence alignment. Numbers at the nodes indicate the bootstrap support based upon 100 resamplings. The PsaA2 and PsaB2 sequences specifically expressed in FRL form distinctive clades with the PsaA and PsaB branches of the tree, respectively.

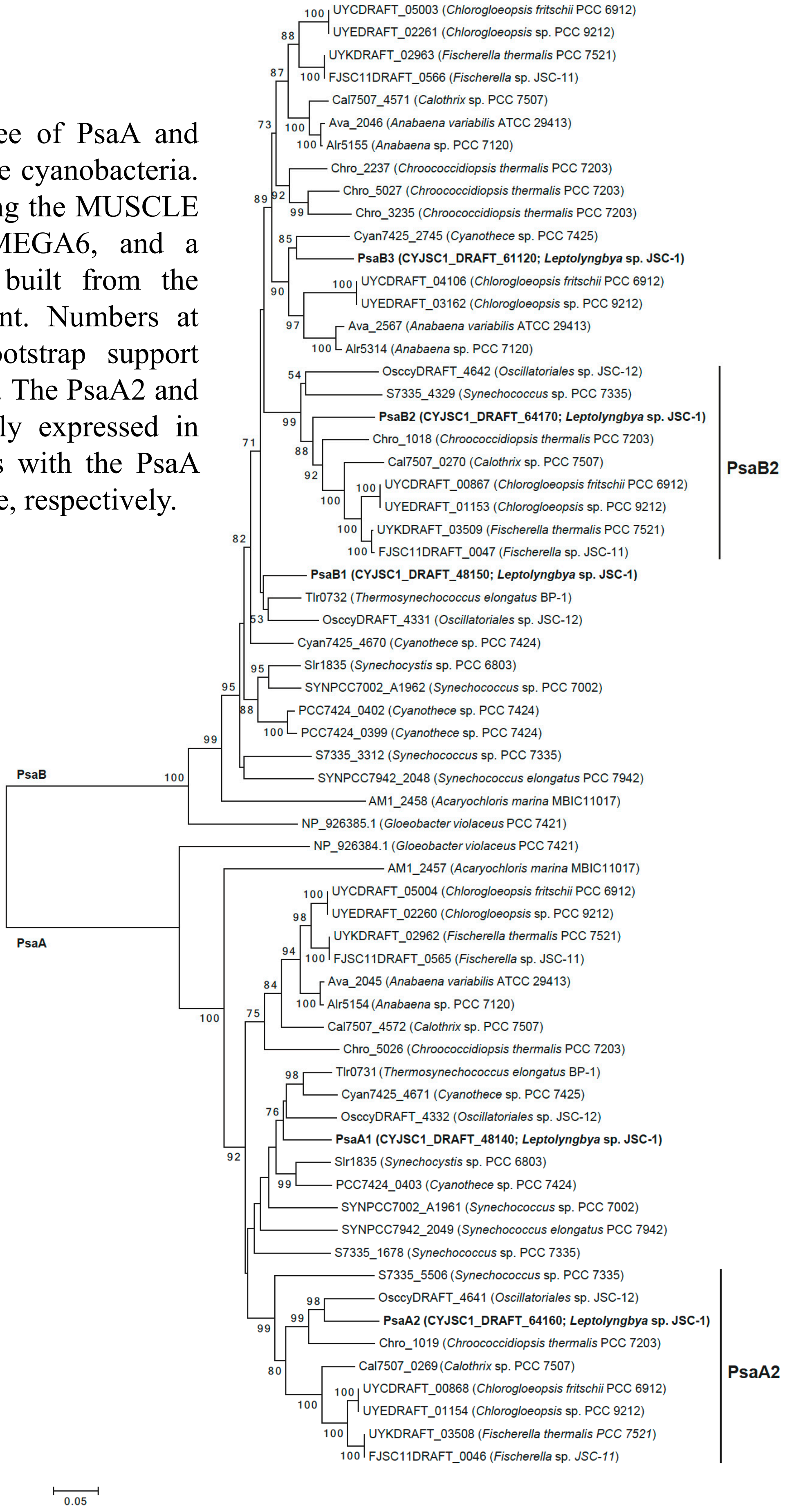

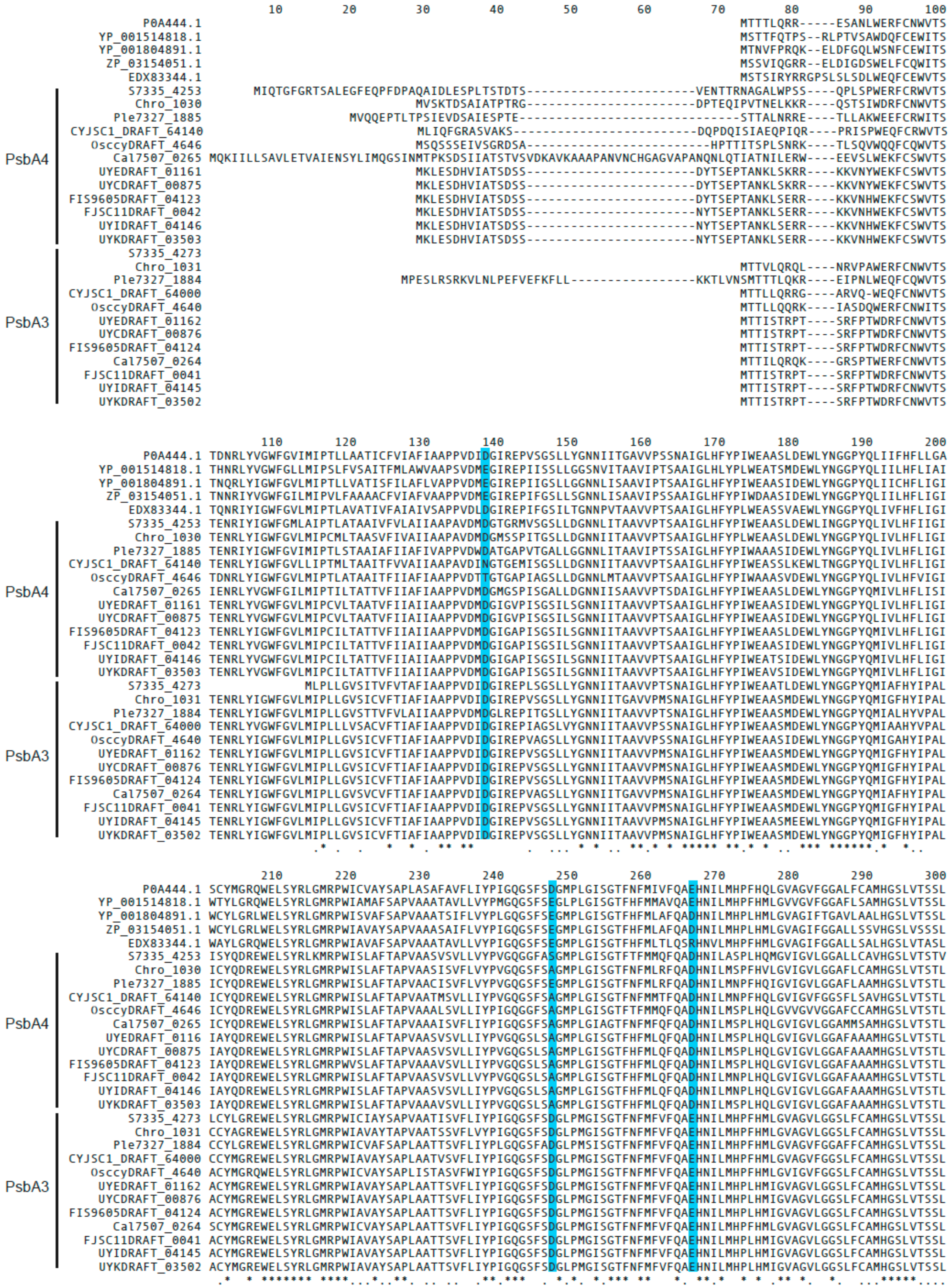

Figure S4. *Cont.*

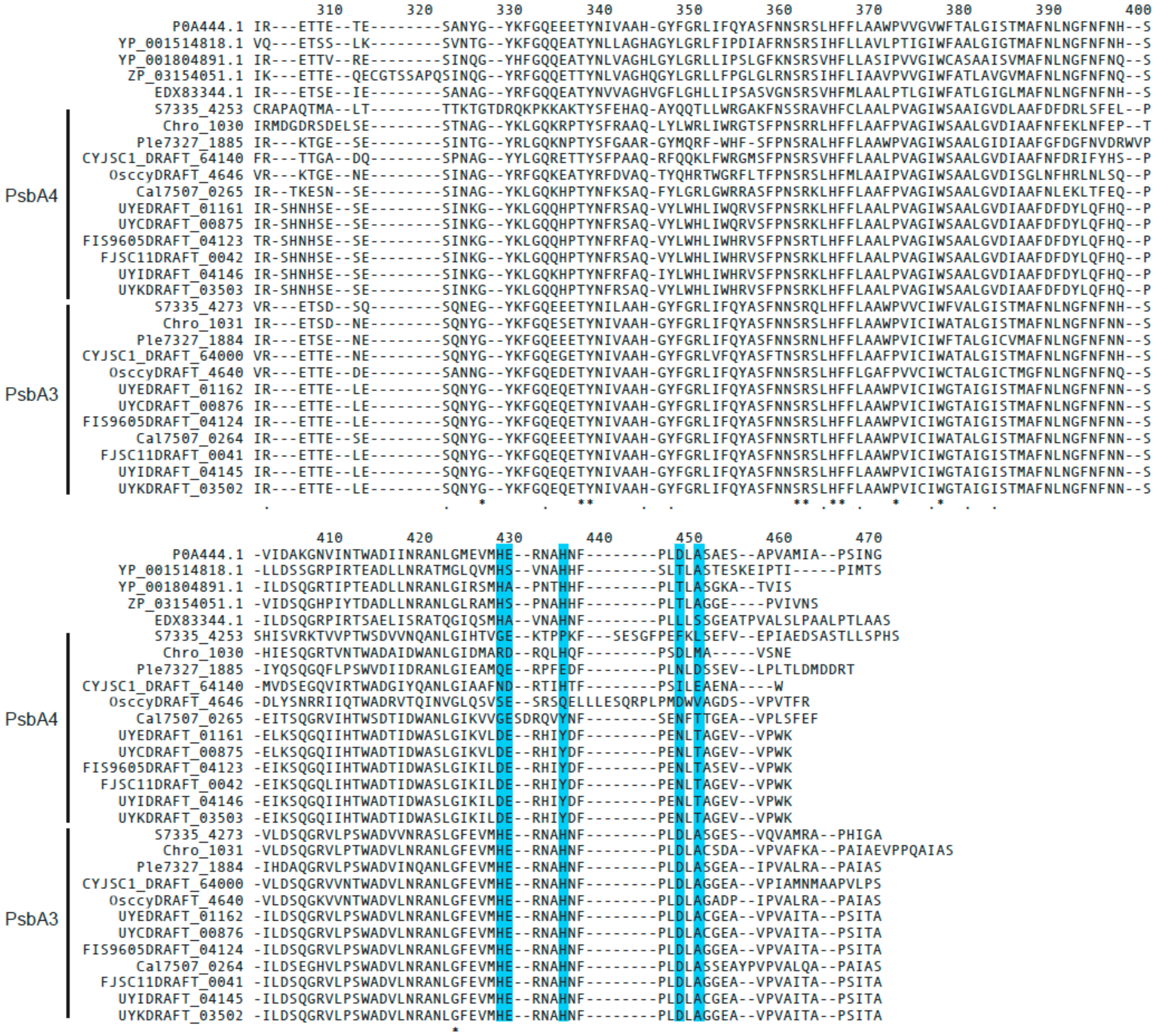

Figure S4. Sequence alignment for PsbA3 and PsbA4 sequences from various strains containing photosynthetic gene clusters similar to that in *Leptolyngbya* JSC-1. A few sequences from other strains are also included for comparison (also see Figure 5). Sequences were aligned using the MUSCLE module implemented in MEGA6. The residues shaded in blue are functionally important residues that provide ligands to important prosthetic groups for electron transfer (for details, see [41]). The D1 protein encoded by *psbA3* includes most of the highly conserved, functionally important residues found in PsbA proteins. The D1 protein encoded by *psbA4* is a “super-rogue” D1 protein that lacks most of the highly conserved, functionally important residues for binding prosthetic groups, in particular the Mn_4 cluster of the oxygen-evolving complex.

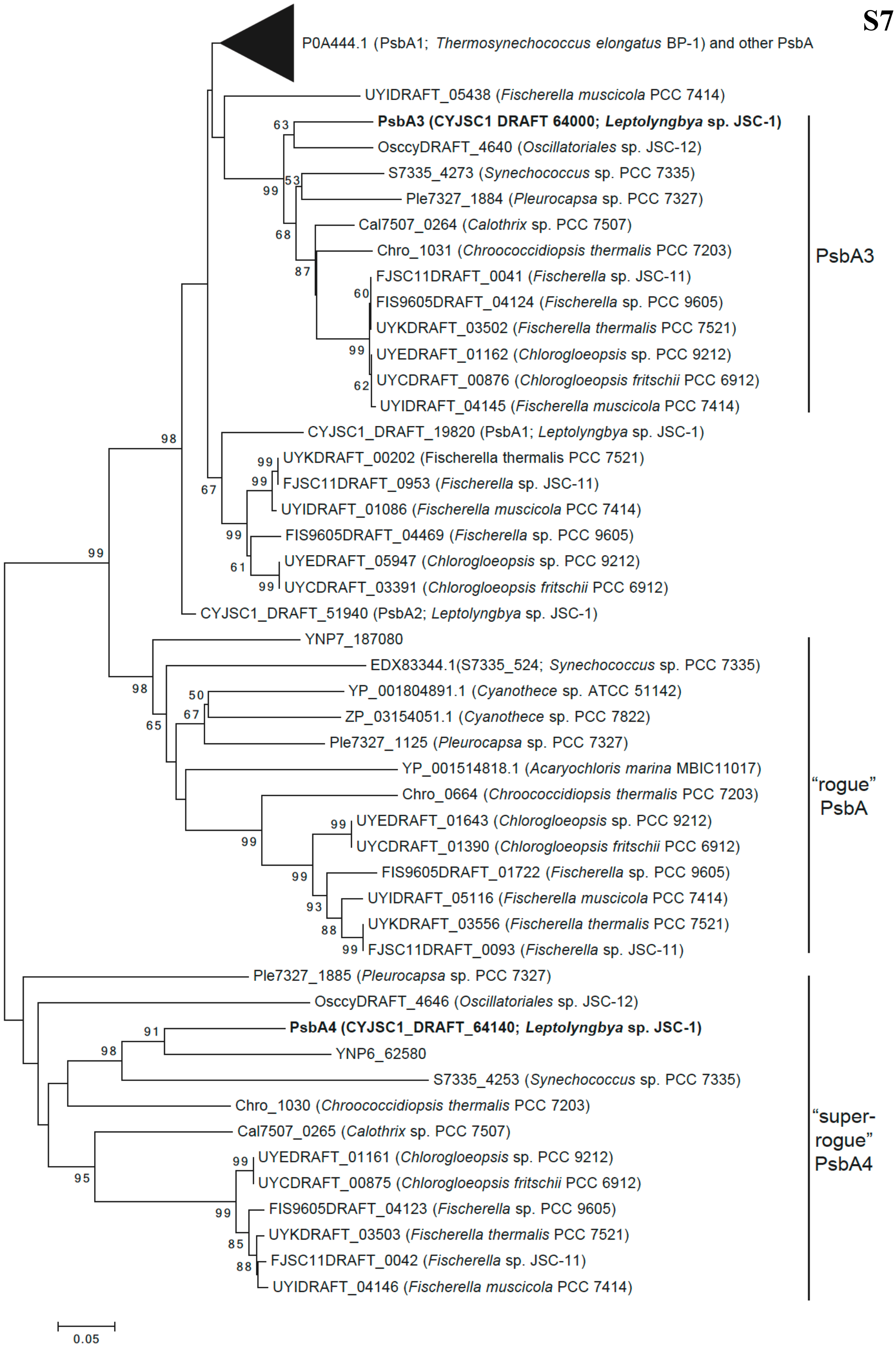

Figure S5. Phylogenetic tree for PsbA1, PsbA3, "rogue" and "super-rogue" PsbA4 sequences. The included PsbA3 and PsbA4 sequences are found in the photosynthetic gene clusters that are similar to the 21-gene cluster found in *Leptolyngbya* JSC-1 [28]. Sequences were aligned using the MUSCLE module implemented in MEGA6. The designations of "rogue" PsbA and "Super-rogue" PsbA sequences are taken from the analysis of Murray [41]. For additional details, see main text.

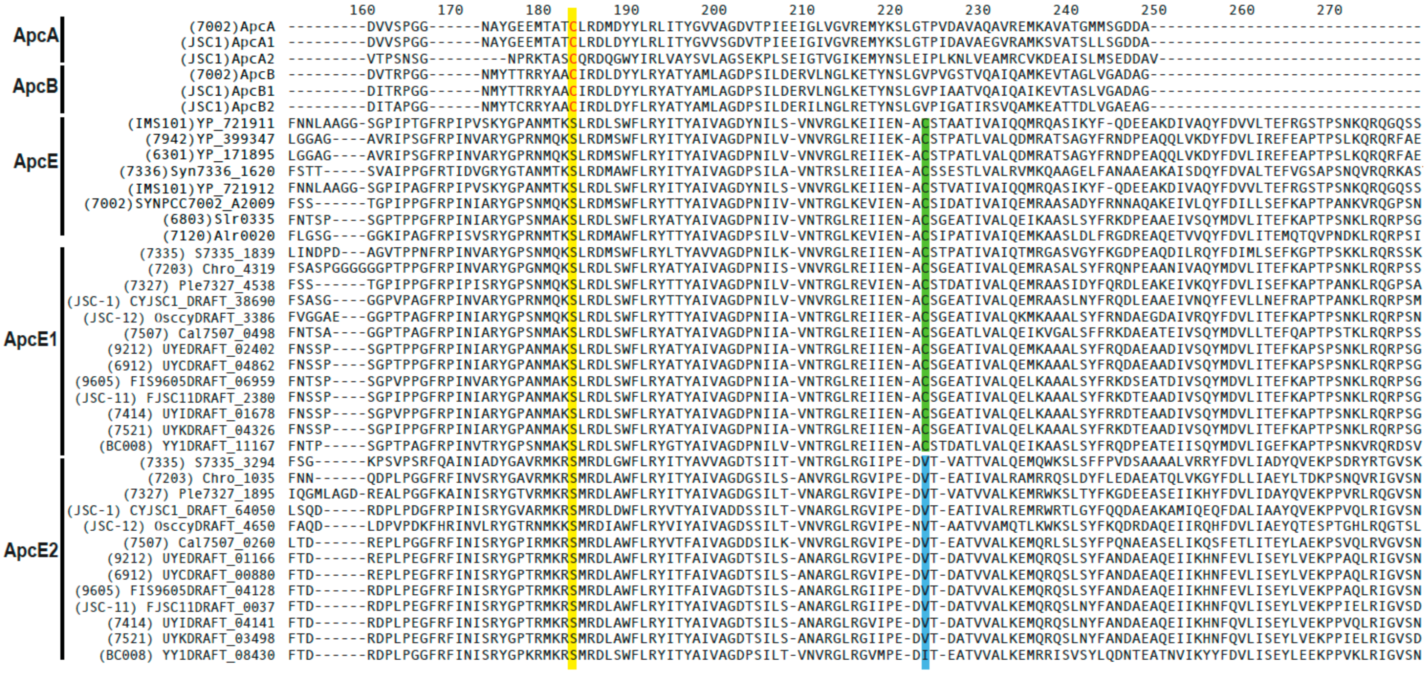

Figure S6. Sequence alignment of a portion of the N-terminal, phycobiliproteins-like domains of ApcE1 and ApcE2 proteins (compared to the corresponding regions of selected ApcA and ApcB sequences). Sequences were aligned using the MUSCLE module implemented in MEGA6. The phycocyanobilin-binding cysteine residue in ApcA and ApcB is highlighted in yellow and red font. The corresponding residue is serine in all ApcE1 and ApcE2 sequences. The chromophore binding cysteine residue for ApcE1 sequences is shaded in green. In all ApcE2 sequences, cysteine does not occur at either position as shown by the yellow and blue shading. Because the chromophore binding pocket is still present (residues 180 to 230), it is probable that the phycocyanobilin associated with ApcE2 is non-covalently bound.

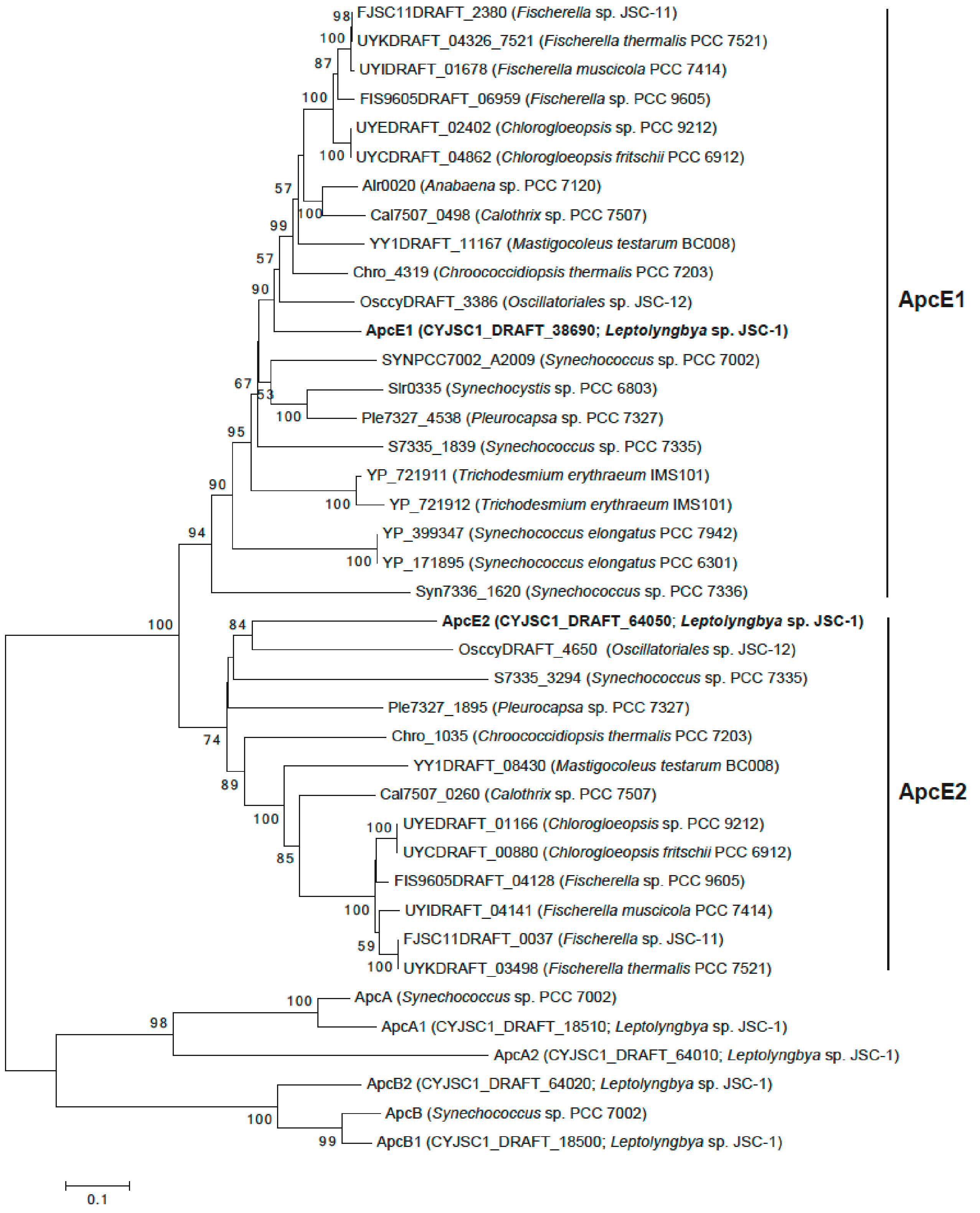

Figure S7. Phylogenetic tree for ApcE1 and ApcE2 sequences from strains with photosynthetic gene clusters similar to that in *Leptolyngbya* JSC-1 and from a few other selected strains with ApcE/ApcE1-like sequences. Sequences were aligned using the MUSCLE module implemented in MEGA6, and a neighbor-joining tree was built from the resulting sequence alignment. Numbers at the nodes indicate the bootstrap support based upon 100 resamplings. Only the N-terminal phycobiliprotein-like domain and REP domains 1 and 2 were included in the analysis. For additional details, see main text.

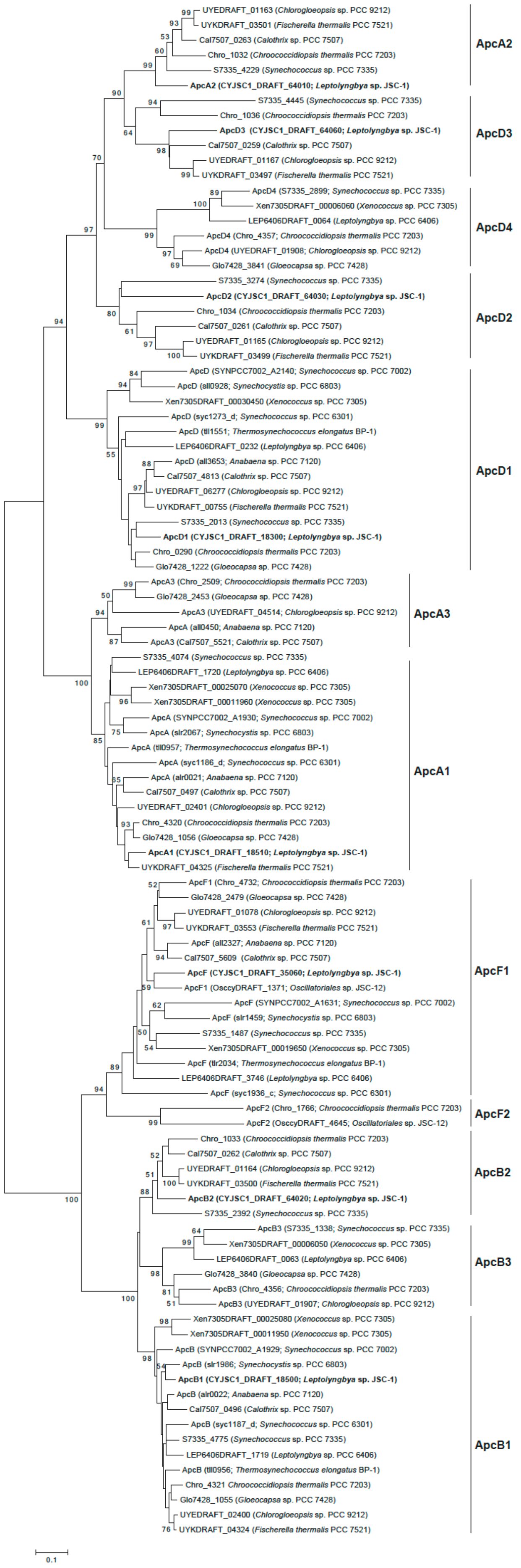

Figure 8. Phylogenetic tree for ApcA, ApcB, ApcD, and ApcF sequences from strains with photosynthetic gene clusters similar to that in *Leptolyngbya* JSC-1 and from a few other selected strains. Sequences were aligned using the MUSCLE module implemented in MEGA6, and a neighbor-joining tree was built from the resulting sequence alignment. Numbers at the nodes indicate the bootstrap support based upon 100 resamplings. Seven clades of allophycocyanin alpha subunits (ApcA1, ApcA2, ApcA3, ApcD1, ApcD2, ApcD3 and ApcD4) were identified. ApcA1 and ApcD1 are replaced by ApcA2, ApcD2, and ApcD3 when cells are grown in FRL. Four clades of beta subunits (ApcB1, ApcB2, ApcB3 and ApcF) were identified. ApcB2 replaces ApcB1 when FaRLiP strains are grown in FRL. In most strains, the *apcF* gene is present as a single-copy gene and presumably the product of that gene is used with both ApcE1 and ApcE2. However, two FaRLiP organisms, *Chr. thermalis* PCC 7203 and *Oscillatoria* sp. JSC-12, have two ApcF genes, and in the latter organism, the *apcF2* gene is localized in the FaRLiP gene cluster (see [28]). The functional roles of ApcA3, ApcB3, and ApcD4 are unknown. Additional details can be found in the main text.

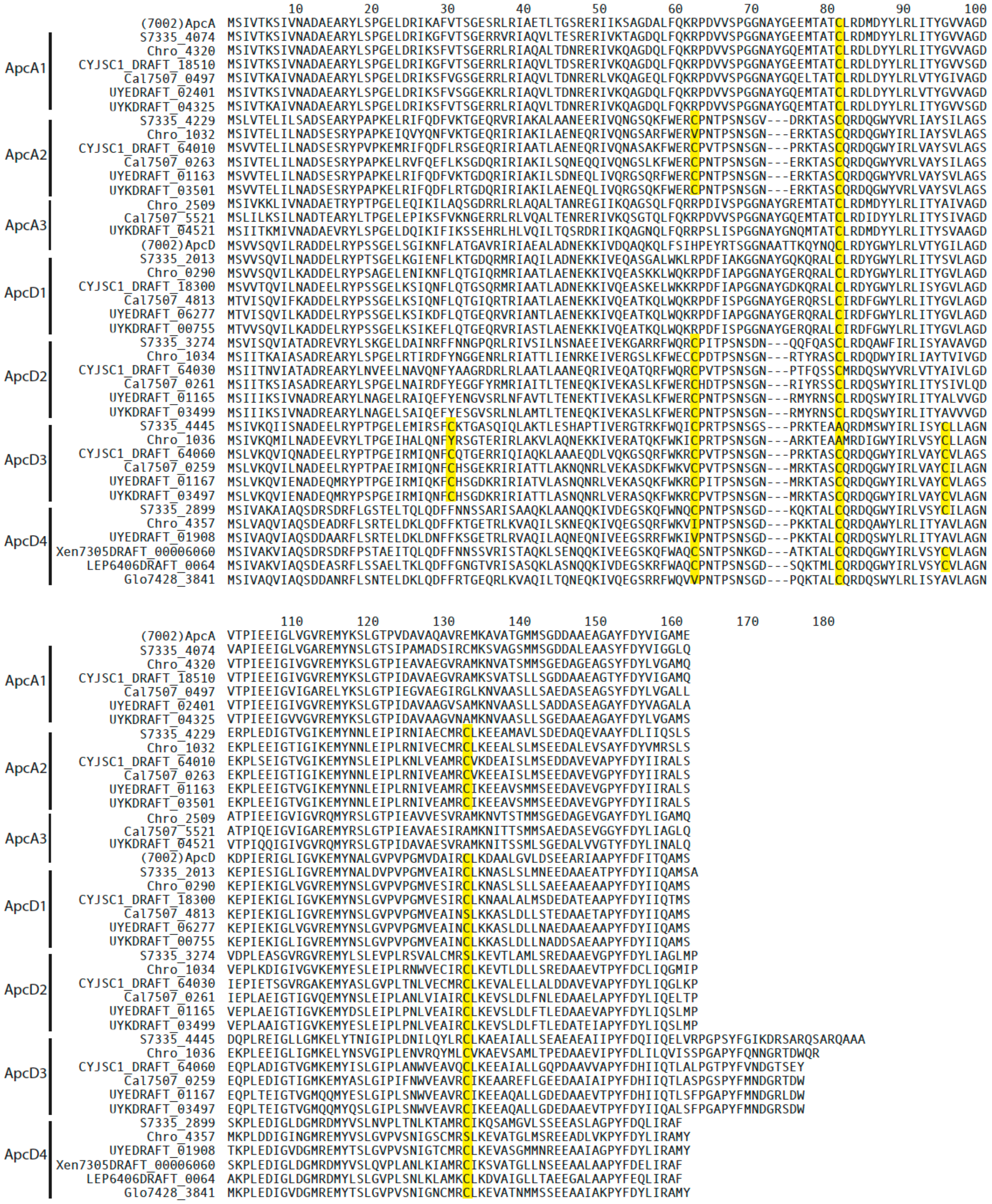

Figure S9. Sequence alignment of ApcA and ApcD allophycocyanin subunits. ApcA1 is the major allophycocyanin alpha subunit produced in WL, GL, and RL, and ApcA2 (ApcD5) is specifically produced when cells are grown in FRL. ApcD1 is the allophycocyanin-B alpha subunit produced in WL, GL, and RL, and ApcD2 and ApcD3 are subunits produced in cells grown in FRL. Sequences were aligned using the MUSCLE module implemented in MEGA6. Yellow shading indicates conserved cysteine residues. The conserved cysteine residue at position 81 typically forms a covalent thioether bond to phycocyanobilin. Additional details can be found in the main text.

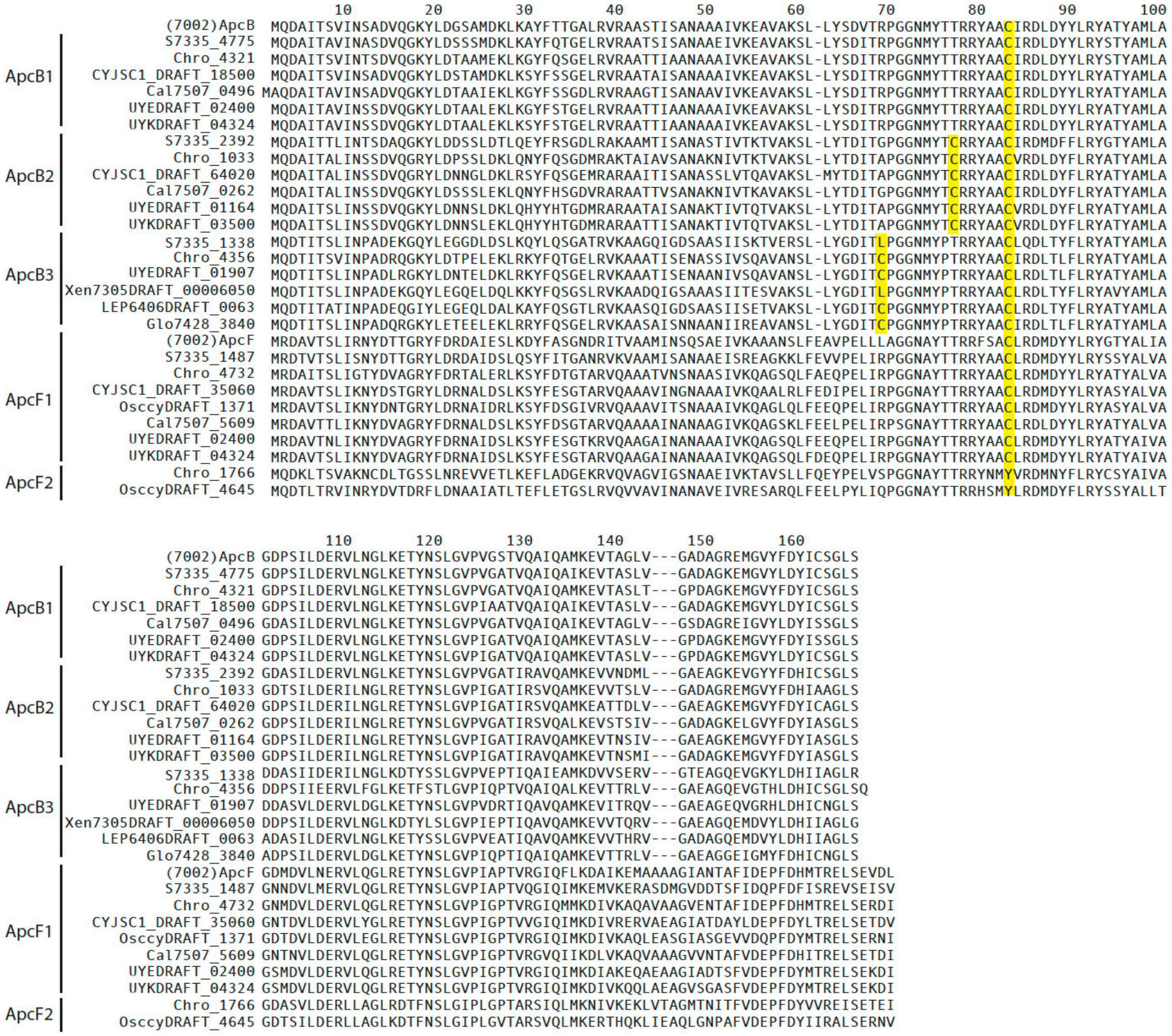

Figure S10. Sequence alignment of ApcB and ApcF subunits of allophycocyanin. ApcB1 is the major allophycocyanin beta subunit produced in WL, GL, or RL, and ApcB2 is the beta subunit of allophycocyanin produced in FRL. In most cyanobacteria, ApcF is a single-copy gene, the product of which apparently functions with both ApcE1 and ApcE2 subunits. Sequences were aligned using the MUSCLE module implemented in MEGA6. Yellow shading indicates conserved cysteine residues. The conserved cysteine residue at position 83 typically forms a covalent thioether bond to phycocyanobilin. Additional details can be found in the main text.
